# ^13^C CP MAS NMR and DFT Studies of 6-Chromanyl Ethereal Derivatives †

**DOI:** 10.3390/molecules27144630

**Published:** 2022-07-20

**Authors:** Piotr Wałejko, Łukasz Szeleszczuk, Dariusz Maciej Pisklak, Sławomir Wojtulewski

**Affiliations:** 1 Faculty of Chemistry, University of Białystok, Ciołkowskiego 1K, 15-245 Białystok, Poland; slawoj@uwb.edu.pl; 2 Department of Physical Chemistry, Chair of Physical Pharmacy and Bioanalysis, Faculty of Pharmacy, Medical University of Warsaw, Banacha 1 Str., 02-093 Warsaw, Poland; dpisklak@wum.edu.pl

**Keywords:** vitamin E, 2,2,5,7,8-pentamethylchroman-6-ol, THP ethers, dynamic ^13^C NMR, GIAO, GIPAW, DFT

## Abstract

Vitamin E consists of a group of compounds including α- β- γ- and δ-tocopherols and α- β- γ- and δ-tocotrienols, containing the chroman-6-ol system. The recognition of the structural and dynamic properties of this system, present in all vitamers, seems to be important for the full explanation of the mechanism of the biological activity of vitamin E. This paper presents results of the structural analysis of the chosen 6-chromanyl ethereal derivatives using experimental (^13^ C NMR-in solution and solid state, as well as variable temperature experiments; single crystal X-ray diffraction) and theoretical (DFT) methods. For one of the studied compounds, 2,2,5,7,8-pentamethyl-6-((tetrahydro-2H-pyran-2-yl)oxy) chroman, the splitting of some signals was observed in the ^13^C dynamic NMR spectra. This observation was explained by the application of a conformational analysis and subsequent DFT optimization, followed by the calculation of NMR properties.

## 1. Introduction

**Figure 1:**
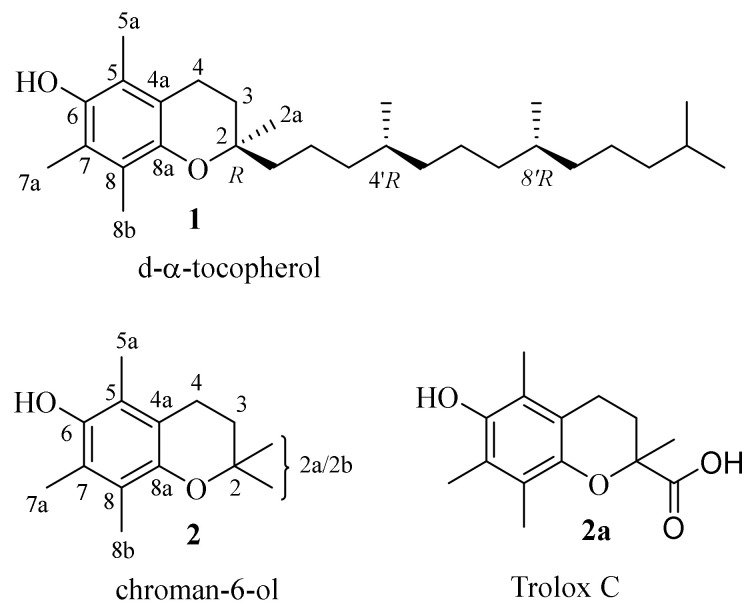
Structures of d-α-tocopherol (**1**), chroman-6-ol (**2**) and Trolox C (**2a**).

Vitamin E (d-α-tocopherol (**1**)) belongs to the group of “so-called” fat-soluble vitamins. Although it has been acknowledged for over a hundred years, it is still an area of interest for chemists and biologists. This is probably due to the fact that vitamin E consists of a group of compounds including α- β- γ- and δ-tocopherols and α- β- γ- and δ-tocotrienols of which the greatest antioxidant and antiradical activity potential, as well as higher abundance in vitamin E natural sources, is possessed by α-tocopherol (**1**, Figure 1). The recognition of structural and dynamic properties of the chroman-6-ol system, present in all vitamers, seems to be an important initial step for the explanation of the mechanism of the biological activity of vitamin E.

Undeniably significant information could be provided from X-ray diffractometric measurements of d-α-tocopherol (**1**). However, due to the flexibility of the phytyl chain (C_16_H_33_) attached to the chroman-6-ol system and amphiphilic properties of the whole molecule, obtaining sufficient quality crystals of **1** for X-ray analysis is very difficult. In the CCDC database, XRD data are available only for d-α-tocopherol succinate [1]. Typically, in the spectroscopic investigation of vitamin E behaviour, the model compounds 2,2,5,7,8-pentamethylchroman-6-ol (**2**) or Trolox C (**2a**, Figure 1) are used. Both compounds **2** and **2a** exhibit the same antioxidant and antiradical properties compared to vitamin E (**1**). They can successfully imitate the action of the d-α-tocopherol molecule in broad areas of structural research and the lower chroman-6-ol (**2**) lipophilicity [2] can help to obtain crystalline derivatives suitable for crystallographic research. The CCDC database contains crystallographic data for **2** [3,4,5,6] and **2a** [7] as well as for their derivatives [8,9,10].

According to the well-established NMR [11,12,13] and ECD [13] data, chroman-6-oles can exist in two main interconverting half-chair conformations. This dynamic process was investigated using the temperature variable ^13^C NMR exploration signals of methyl groups C-2a/2b in **2**, and the C-2a signal in α-tocopherol esters (acetates, benzoates, nicotinates, etc.). The observed temperature dependence signal separation allowed for the determination of the dihydropyran ring pseudorotation barrier. The Gibbs free energy of the activation (Δ*G^#^*) of the process was successfully achieved using ^13^C NMR techniques in a solution [13,14] and solid state [8,15,16]. The resulting data regarding the α-tocopherol nicotinate, acetate and succinate indicated that for interconverted methyl groups (2a and 2b) the coalescence temperature (*T_c_*) occurs at 304, 296 and 288K with Δ*G^#^* values of 62.6, 61.0 and 58.0 kJ mol^−1^, respectively [17]. In the case of chromanol-6-ol acetate, the calculated values were slightly lower and reached *T_c_* = 293K and Δ*G^#^* = 59.5 kJ mol^−1^ [12,14]. 

**Figure 2:**
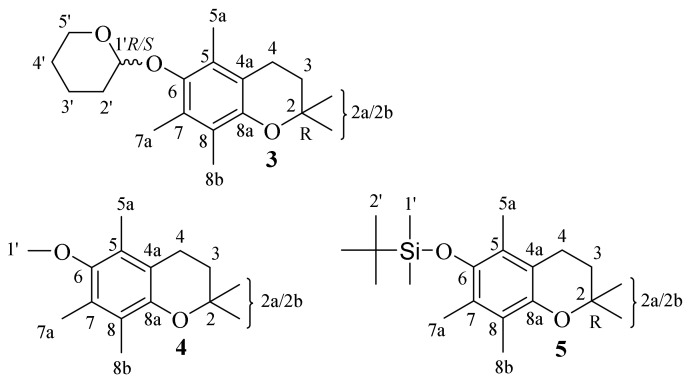
The examined compounds **3**–**5 c**hemical structures. The carbon numbering was used accordingly to IUPAC rules [18,19].

As a contribution to this field of research, we presented current results on the conformational analysis of chroman-6-ol esters using X-ray data and NMR techniques (solid-state, solution and dynamic NMR) supported by density functional theory (DFT) calculations. The aim of this work was to study the effects that arise in the pentamethylchroman-6-ol system after C-6 hydroxyl group etherification, especially for the conformational labile tetrahydropyran residue (**3**). The chemical structures of the investigated compounds are shown in Figure 2.

In this paper, the X-ray structure of ethereal chroman-6-ol derivatives is presented for the first time, e.g., 2,2,5,7,8-pentamethyl-6-((tetrahydro-2*H*-pyran-2-yl)oxy) chroman (chroman-6-ol, **3**), 6-methoxy-2,2,5,7,8-pentamethylchromane (**4**), and *tert*-butyldimethyl((2,2,5,7,8-pentamethylchroman-6-yl)oxy)silane (**5**). To the best of our knowledge, in the existing scientific literature there exists no information on the dynamic effects in this type of ethereal chroman-6-ol derivative. 

## 2. Results and Discussion

The XRD data for vitamin E as well as its derivatives are available only for tocopherol succinate [1]. Generally, the available XRD data were recorded for vitamin E model compounds: 2,2,5,7,8-pentamethylchroman-6-ol (**2**) (MOPHLB) [3,4,5,6] and Trolox C (DEWVOQ02) [7] as well as for its derivatives [8,9,10]. In the present work, the new X-ray structures of chroman-6-ol ethers, including THP **3**, methyl **4** and silyl **5** were presented. The ethers **3**–**5** crystalize in the I2/a, P2_1_/c and P2_1_/n space group, respectively, with the unit cell containing sixteen molecules in **3**, four in **4** and eight in **5** (Table 1, for graphical presentation, see Figures S1–S3). The asymmetric part of **3** and **5** contains two independent molecules (denoted as molecules A and B) while the asymmetric part of **4** forms one molecule. The selected chroman-6-ol ring’s bond lengths (Å), bond angles (°) and torsion angles (°) in **2**, **4** and **5** were obtained using the X-ray data which are presented in Tables S1–S3.

The differences in the C-C bond length between methyl ethers **4, 3** and **5** were generally below the values of estimated uncertainty 3σ (Table S4). However, in silyl ether **5** the lengthening of C6-C7 and shortening of the O2-C6 bond in contrast to **3** and **4** was observed. The bond O2-C6 in **5** was 1.39 Å, while in **3** and **4** it was 1.40 Å. The interesting information on the chroman-6-ol system provided a comparison of the aryl bond length in esters **3**, **4** and **5** with a similar XRD structure of 4-methoxy-2,3,5,6-tetramethylphenol (4MTP, CCDC MOPHLA) (Table S5). In the ethers, the bonds C8a-C4a, C4a-C5, C6-C7 and C8-C8a were noticeably longer (over 0.1 Å), while the ethereal bond (C8a-O1) was shorter by c.a. 0.3 Å. The observed differences indicated the higher deformation of the aromatic ring in chroman-6-ol ethers (**3**, **4** and **5**) compared to those observed in unsubstituted phenol or 4MTP. A similar conclusion was provided in an analysis of the aromatic ring internal bonds angles (Table S2) and dihedral angles (Table S3). The *ipso*-C6 and C8-8a-4a angle in ethers **3**, **4** and **5** were higher by 1.6–2.5° compared to unsubstituted benzene, while the other angles were reduced by 0.8–1.0°. The dihedral angles C8-C8a-C4a-C5, C4a-C5-C6-C7 and C5-C6-C7-C8 reached up to 5–6° which indicated significant aromatic ring deviation from planarity.

**Figure 3:**
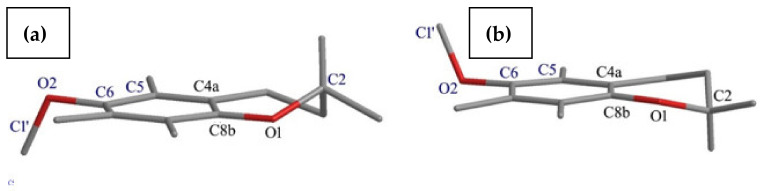
The main heterocyclic ring conformers in chroman-6-oles: 2-*exo*-3-*endo* (**a**) and 2-*endo*-3-*exo* (**b**) (dihedral angles *θ* and *γ* were denoted as C4a-C8a-O1-C2 and C5-C6-O2-C1′, respectively).

The NMR [11,12] and ECD [13] data proved that in solutions the chroman-6-oles mainly occur in the following two interconverting half-chairs conformations: 2-*exo*-3-*endo* and 2-*endo*-3-*exo* (depicted in Figure 3). In the case of chroman-6-ol ethers (**3**, **4** and **5**), the examination of crystal unit packing indicated the presence of both in crystals, and half-chair conformations characterized by a dihedral angle *θ* (denoted as C4a-C8a-O1-C2). As a consequence, in the crystal lattice of **3**, **4** and **5** the 2-*exo*-3-*endo* and 2-*endo*-3-*exo* conformations of heterocycle ring in a ratio of 1:1 was observed (they have the same *θ* value with opposite notation).

The flanking 5a and 7a methyl group’s steric hindrance in the analyzed derivatives hampered rotation residue at C-6. As a consequence, the methyl-, THP- or TBDMS- pendant groups twisted toward the average chroman ring plane and the twisting level was characterized by the value of the torsion angle *γ* (denoted as C5-C6-O2-C1′/Si). 

**Figure 4:**
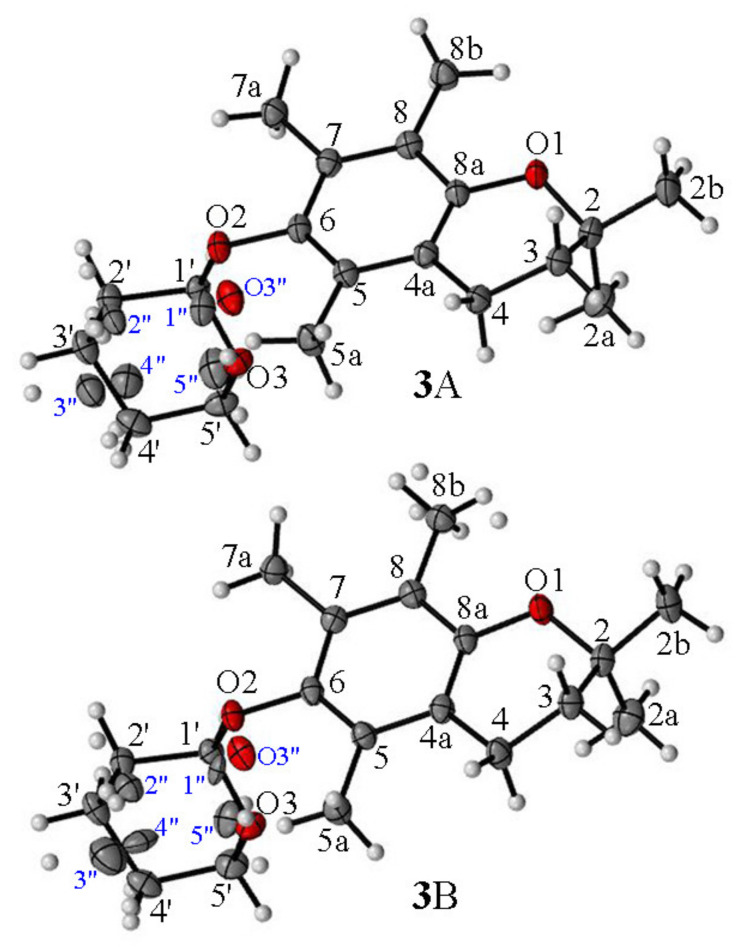
The asymmetric units of ether **3** and the atom-labelling scheme in molecules **3**A and **3**B. Displacement ellipsoids were drawn at the 50% probability level.

According to the obtained XRD data, the asymmetric unit of **3** contained two independent molecules in a 2-*exo-*3-*endo* conformation **3**A (*γ* = −103.9°, *θ* = 16.9°) and **3**B (*γ* = −105,0°, *θ* = 14.2°) (Figure 4). In contrast to **4** and **5,** in all molecules of **3** the THP residue and *axial* methyl group (C2) were in *cis* relation. The XDR data indicated an almost perpendicular location of the THP ring concerning the average chroman-6-ol plane with the oxygen atom O3 pointed toward the aromatic ring. As a consequence, the intermolecular distance (C2a^…^O3) in molecule **3**A reached 6.30 Å or 6.55 Å (C2aA^…^O3A/O3”A) and in **3**B it reached 6.76 Å or 6.89 Å (C2aB^…^O3B/O3”B). 

**Figure 5:**
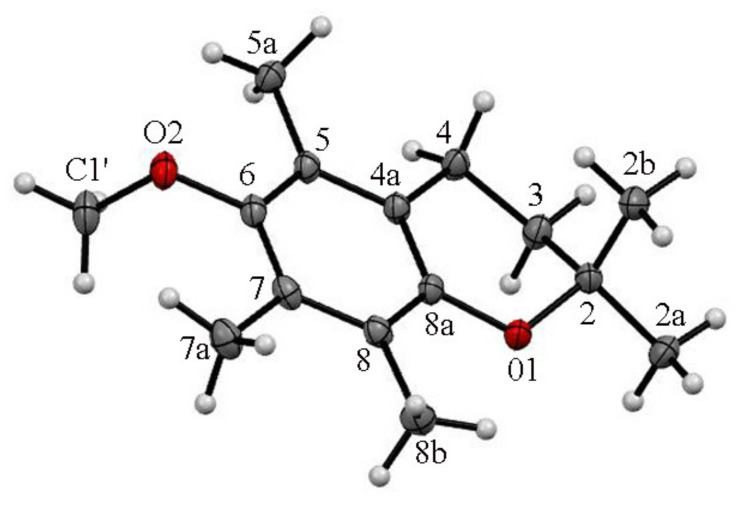
The asymmetric units of ether **4** and the atom-labelling scheme. Displacement ellipsoids were drawn at the 50% probability level.

According to the obtained XRD data, the asymmetric unit of **4** concerning the molecule in 2-*endo-*3*-exo* conformation *was* characterized by a dihedral angle *θ* and *γ* values of +21.3° and −91.0°, respectively. Conversely, in the crystal lattice of **4** equal a symmetrically identical structure in a 2-*exo*-3-*endo* conformation (*γ* = +91.0° and *θ* = +21.3°) was observed. In both conformers the methoxy residue and *axial* methyl group (C2) were *trans-*oriented concerning the average chroman-6-ol plane with a distance of C2b^…^C1′ = 7.548 Å. In crystals with a short contact interaction (H7a^…^C6 c.a. 2.85 Å), they formed dimeric structures translated along the b axis forming columns of **4** (Figure 5).

**Figure 6:**
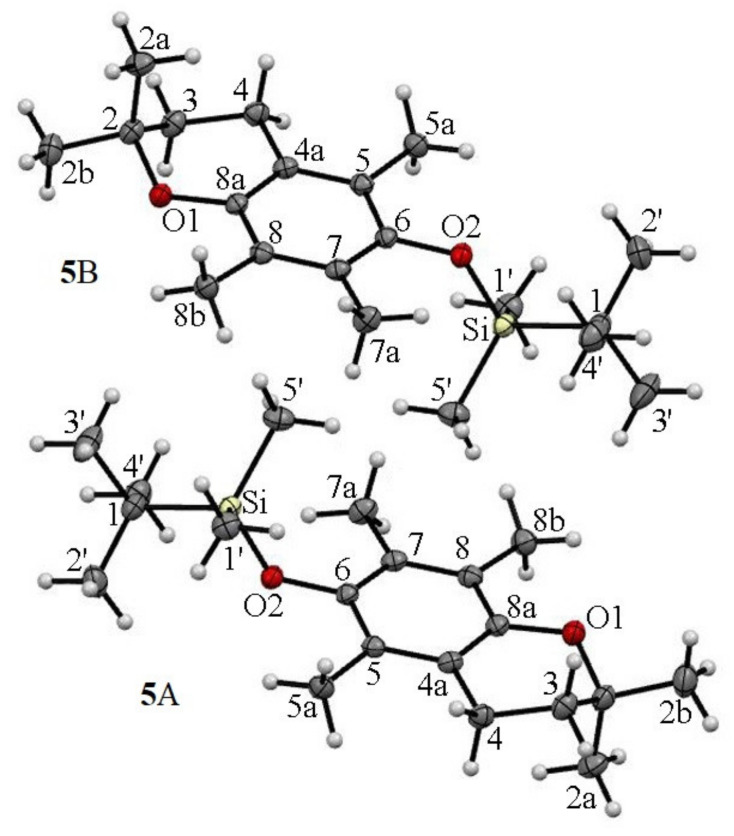
The asymmetric units of ether **5** and the atom-labelling scheme in molecules **5**A and **5**B. Displacement ellipsoids were drawn at the 50% probability level.

The asymmetric unit of silyl ether **5** contains two independent molecules **5**A (2-*exo-*3*-endo*) characterized by angles *γ* and *θ* values of +94.8° and +19.7°, respectively, and molecule **5**B in a conformation of 2-*endo-*3*-exo* with *γ* = −94.7° and *θ* = −18.5°. Apart from the conformers depicted in Figure 6, the crystal lattice of **5** contained an equal amount (1:1) of rotamers with an opposite heterocycle ring conformation of 2-*endo-*3*-exo* with *γ* *= −*94.8° and *θ* = −19.7° and of 2-*exo-*3*-endo* with *γ* = +94.7° and *θ* = +18.5° (Figure S3, See Supplementary Materials). In all molecules, the TBDMS- moiety and *axial* methyl group (C2a) were in *trans* relation to the average chroman-6-ol plane. The crystal structure of **5** was stabilized by the presence of C^…^H and O^…^H type short contacts (e.g., O2A^…^H2aB = 2.394 Å; O2B…H2aA = 2.401 Å) presented in Figure S4. 

### 2.1. ^1^H and ^13^C NMR

The ^1^H and ^13^C NMR data for α-tocopherol (**1**), and chroman-6-ol (**2**) are well documented in the literature [1,8,9,12,13,14,20,21] while the NMR data concerning the α-tocopherol alkyl ethers are relatively scarce. They were only reported by Urano et al. as the main product of the radical scavenging reaction of α-tocopherol with some alkyl radicals [22,23]. The ether **3** ^1^H and ^13^C NMR full signal assignment, in solution, were performed with the aid of 2D NMR shift correlation experiments (DQF-COSY, HSQC and HMBC). The three bond coupling (^3^*J*_H-H_) on DQF-COSY for **3** allowed for full proton signal assignment, assuming that the most deshielded signal (4.67 ppm) was attributable to H1′. The HMBC spectrum showed sets of cross-peaks (^2−3^*J*_H4-C_) between H4 and C4a, and C5 and C8a which allowed for the determination of signals C4a (117.04 ppm), C5 (126.38 ppm) and C8a (148.00 ppm). The signals C5a and C8b were identified by the presence of a cross-peak between C5 and H5a (^2^*J*_C5-H5a_) and C8a and H8b (^3^*J*_C8a-H8b_), while the proton H7a signal was determined due to the presence of a cross-peak between carbon C6 and protons H5a and H7a. Similarly, the 2D NMR experiments allowed for full assignment in the ^1^H and ^13^C NMR spectra of ethers **4** and **5**. 

### 2.2. ^13^C CP MAS NMR

**Figure 7:**
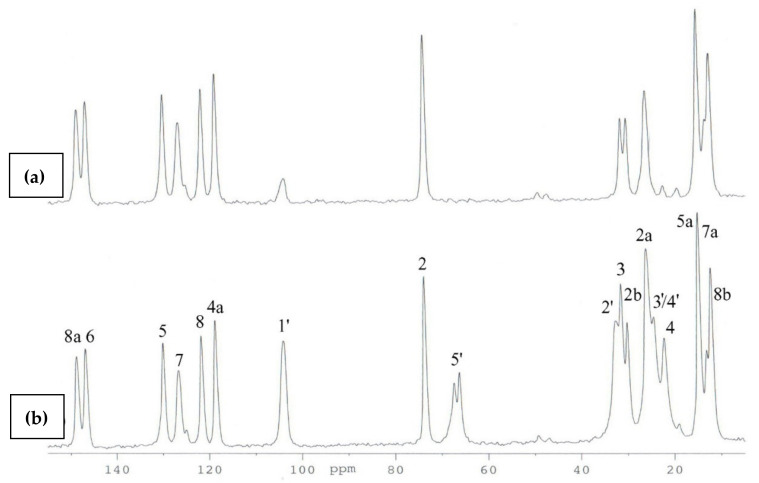
^13^C CP MAS NMR of **3**: (**a**) with DD pulse sequence, (**b**) standard spectra.

The use of the DD technique made it possible to identify the signals originating from the groups of quaternary carbons (C2, C4a, C5, C6, C7, C8, C8a), and at the same time, all signals from the methyl groups (C2a, C2b, C5a, C7a, C8b) were visible in this spectrum (Figure 7). This indicated the high rotational dynamics of these groups in the crystal. Simultaneously, in the ^13^C CP MAS NMR spectra of **3**, the splitting of some signals was observed. We assumed that this was caused by the presence of two molecules in the asymmetric unit cell (Z’ = 2). Therefore, to test this hypothesis, periodic DFT calculations were performed. The calculated NMR chemical shifts (Table 2) provided unambiguous signal assignment. The large differences between the chemical shifts, both experimental and theoretical, of the corresponding atoms of two inequivalent molecules of **3** were found for carbon atoms 2b, 3, 5a, 4′ and 5′. DFT calculations made it possible to unambiguously assign signals in the ^13^C CP MAS NMR spectrum A and at the same time allowed us to distinguish signals of C2a and C2b methyl carbons in the ^13^C CP MAS spectrum. Both groups had different orientations in relation to the chromanol plane. In the case of the C2b methyl group parallel to the chromanol plane, the signal was deshielded and split due to magnetic inequality in both molecules in the crystal cell, and in the case of the C2a carbon perpendicular to the plane, the signal was shifted towards the higher field and signal doubling was observable, which was in agreement with the DFT calculation for the crystal structure.

### 2.3. C DNMR Study

According to the literature, the Gibbs free energy of activation Δ*G*^#^ estimation was taken as a measure of the barrier height of bond rotation. The reported Δ*G*^#^ values in esters of α-tocopherol (**1**), or chroman-6-ol (**2**) were in the range of 59–63 kJ mol^−1^ at *T_C_* ca. 300K. The 6-amino- and 6-nitro-2,2,5,7,8-pentamethylchroman conformational analysis indicated a significant effect of the electro- donor or electro-acceptor properties of the C6 moiety on the kinetics of heterocyclic ring interconversion [9], while for free α-tocopherol (**1**) or chroman-6-ol (**2**), the coalescence point was not achieved even at the lower temperature measurement (ca. 160K). 

To the best of our knowledge, there are no reports on the influences of alkyl or silyl moiety in chroman-6-ol ethers **3**–**5** on the heterocyclic ring interconversion. For compounds **3**–**5,** the temperature-dependent ^13^C NMR (DNMR) spectra were recorded in the temperature range of 295 K**–**165 K. In contrast to the α-tocopherol (**1**), or chroman-6-ol (**2**) esters, ethers **3**–**5** did not show any significant dynamic effects on the resonances of the methyl group C2a and C2b. Only a small broadening of merged C2a/2b resonance was observed with signal half-width ca. 12 Hz (**5**) and 42 Hz (**4**) (Figures S5 and S6). The temperature-dependent spectra indicated that the heterocyclic ring barrier interconversion in the chroman-6-ol ethers **3**–**5** was significantly smaller compared to that reported in α-tocopherol (**1**) esters and comparable with free α-tocopherol (**1**) or chroman-6-ol (**2**). 

The carbon spectra of ether **3** showed two separated methyl signals at 26.86 and 26.80 perpendicular ppm which were attributed to diastereotopic methyl groups C2a and C-2, one in a parallel and the second one in a perpendicular orientation with respect to the average chroman-6-ol plane. The temperature dependence ^13^C NMR spectra showed that C2a and C2b carbon signals diminish intensity with a decreasing temperature, forming one broadened signal at ca. 180 K (Figure S7).

**Figure 8:**
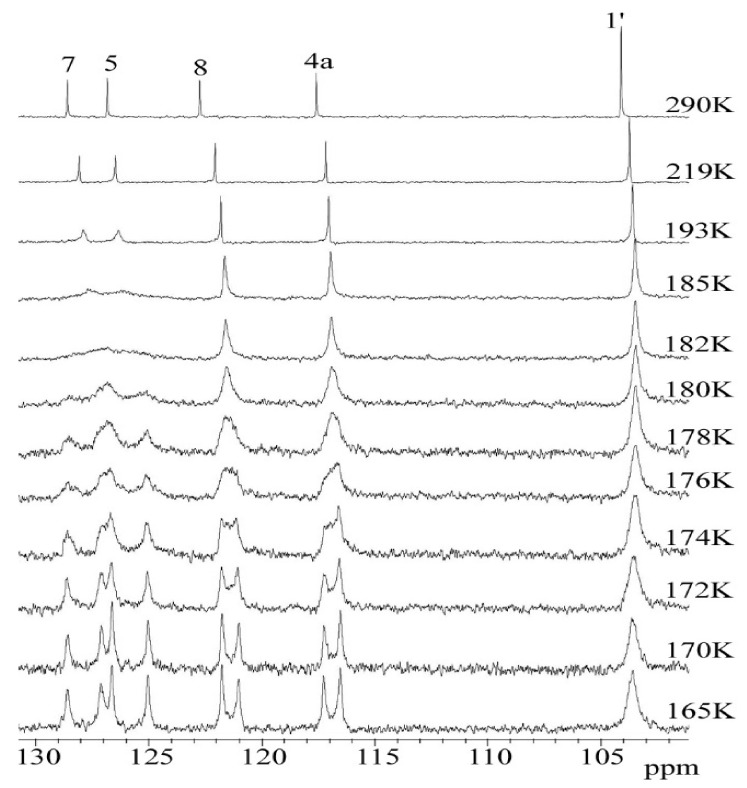
Temperature variable ^13^C DNMR spectra of **3** in temp range 290–165 K. The full-scale spectra (0–170 ppm) were presented in Figure S7.

However, in the ^13^C DNMR spectra of the THP ether (**3**), the significant temperature dependence of the aromatic carbon signals’ shape and intensity was observed. The ^13^C NMR spectra of **3** recorded at a temperature above 219 K showed sharp resonances of aryl carbons (C5 and C7) as well as the methyl group C5a and C7a. In the low-temperature region, these signals gradually disappeared and below 175 K two separated signals for each of them were observed (Figure 8). 

**Figure 9:**
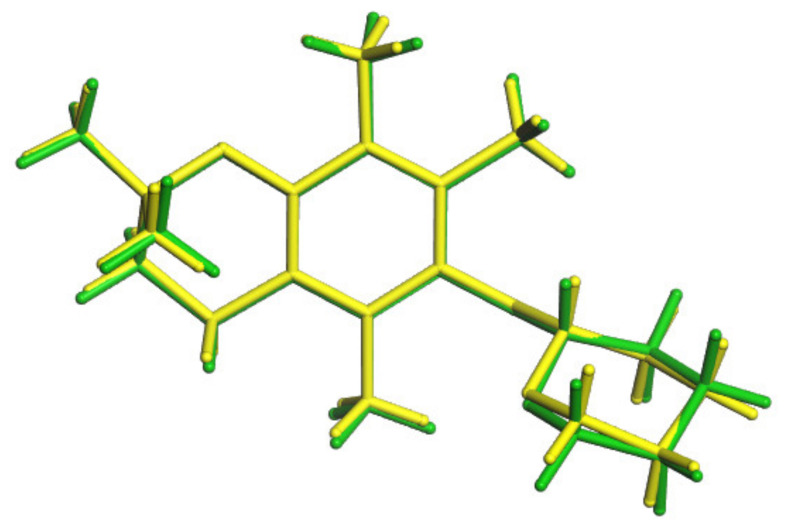
Comparison of conformations of **3**; **3a** is the green one, conformation from SCXRD is the yellow one.

To explain the changes occurring at the molecular level after lowering the experimental temperature and the signal splitting observed in the ^13^C DNMR spectra, a conformational search of **3** was conducted. The 26 different conformations obtained at this stage were optimized at the DFT level and NMR property calculations were performed for each of them. After this step, it was found that the initial twenty six structures converged into six different ones (Table S7). The best agreement between the calculated and experimental (at 290 K) ^13^C chemical shifts was found for the lowest energy conformation, namely **3a** (Table 3). This conformation was also very similar to the one present in the crystal structure of **3** (Figure 9). 

The absolute values of differences between the experimental and calculated chemical shift values (Δ) for **3a** did not exceed 2.5 ppm, which is considered to be accurate. The next two conformations, in the order of increasing energy, (**3f**, **3g**) were discarded as the Δ for some atoms exceeded 4.5 ppm, reaching even 6.39 ppm in one case. The largest values of Δ were observed for the atoms forming a tetrahydropyran ring as well as for C5. 

The subsequent conformation, in order of increasing energy–**3h** was identified as one of the signals appearing in the ^13^C DNMR spectra of **3** below 180 K. The largest differences in the chemical shift values between the corresponding atoms of **3a** and **3h** molecules, exceeding 1 ppm, were found for atoms C4a, C5, C7, C8 and C1′, which was in perfect agreement with the experimental results; as for the signals of those atoms, splitting was observed in the ^13^C DNMR experiments. The remaining conformations were not only energetically less favourable but also significant differences between the calculated and experimental chemical shifts were found for them. For example, in the case of **3i** the Δ for C2′ and C3′ exceeded 7 ppm while for 5′ it reached almost 9 ppm. For **3k**, the Δ for C3′ and C4′ exceeded 4.5 ppm while for **3u** the Δ for C7 and C4′ exceeded 5.5 ppm. 

**Figure 10:**
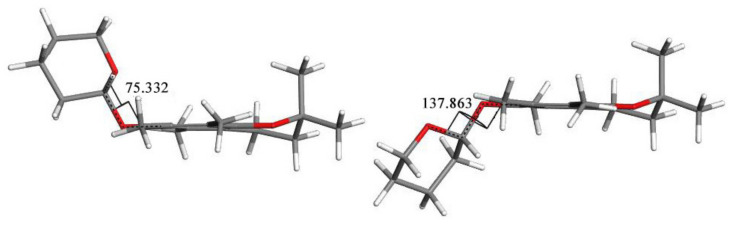
Conformations of **3**: a (**left**) and h (**right**). The C6-O-C1′-O torsion angle values are 75.332 for **3a** and 137.863^o^ for **3h**, respectively.

A comparison of the structures of **3a** and **3h** revealed that the major difference between those two conformations was the C6-O-C1′-O torsion angle value, of 75.332^o^ for **3a** and 137.863 for **3h**, respectively (Figure 10). In **3a,** the tetrahydropyran ring and C2a methyl group were located on the same side of the chroman-6-ol ring while in **3h** they were on the opposite sides. 

Based on a set of ^13^C NMR spectra recorded at different temperatures (Figure 8), the free energy of the activation barrier Δ*G^#^* was assigned by simply finding the coalescence temperature (*T_c_*) and Δν_(max)_ values (maximum signal separation). According to the literature [24,25,26] for the coalescence temperature (*T_c_*) at which the dynamic process occurs, the exchange rate is given by Equation (**1**).

*k*_c_ = π/Δν/√2 (1)

The rotation barrier at *T_c_* was determined in the temperature range of 219–165K in CD_2_Cl_2_ solutions, with steps ca. 2K for carbon atoms C5, C7, C8, C4a and methyl C7a and C5a. The Δν_(max)_ values were determined based on the differences in the chemical shift of the analyzed carbon atom at the lowest accessible temperature. The estimation of free energy of the activation barrier Δ*G^#^* was achieved using Equation (**2**) [25].

Δ*G^#^* = 1.914.10^−2^ *T_c_* [9.972 + log(*T_c_*/Δν_(max)_)] (2)

ΔThe preliminary calculations of Δ*G*^#^ values for carbon signals C-7, C-5, C-8 and 4a and methyl C5a and C7a are presented in Table 4.

**Figure 11:**
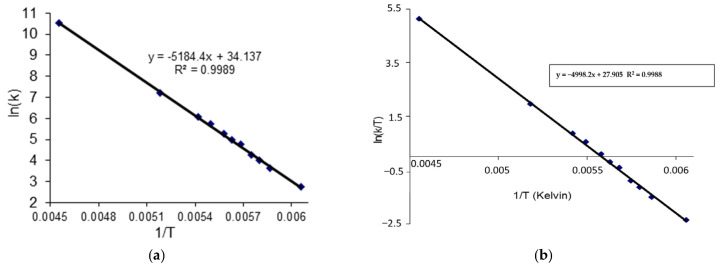
The *Arhenius* (**a**) and *Eyring* (**b**) plots.

The second approach to the complete line analysis (CLA) method was used to calculate the exchange rate constants (*k*) and the other kinetic parameters could finally be determined using the Arrhenius (**3**) and Eyring (**4**) equations (Figure 11).

*k* = A*e^−Ea/RT^* (3)

*k = κ · k_b_T/h · e^−^*^*G#/RT*^ (4)

**Figure 12:**
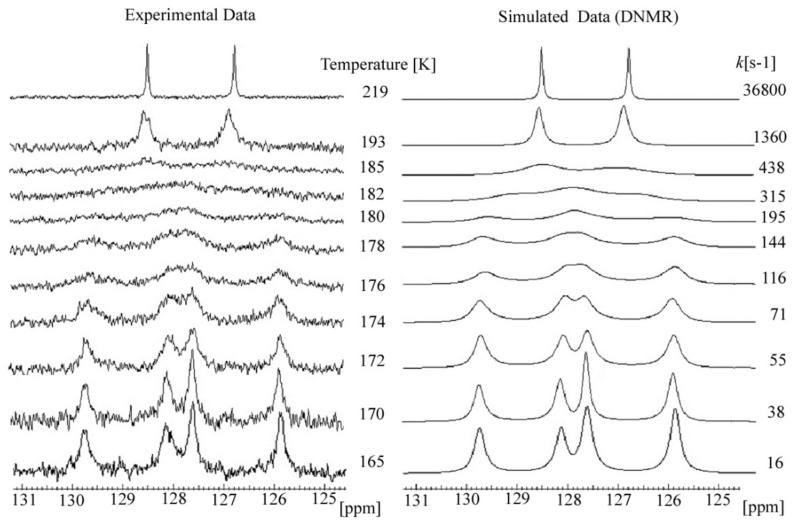
Experimental and simulated variable temperature ^13^C NMR spectra resulted for ether **3** (temp. range 165–219 K).

The ^13^C dynamic NMR spectra (Figure 12) were simulated using Reich H.J. WinDNMR free-shareware [27]. The exchange rate constants (*k*) at each temperature were calculated using the function implemented in WinDNMR software. The simulated data were subsequently used for the *Arrhenius* and *Eyring* plots (ln *k* = *f*(1/T) and (ln *k/*T = *f*(1/T)), respectively (Figure 11). The kinetic parameters, i.e., enthalpy (Δ*H*^#^), entropy (Δ*S*^#^) and energy of activation (*Ea*) were estimated from the related slopes and intercepts of these plots.

The average Δ*G*_1_*^#^* value calculated using equation **b** was 35.10 kJ mol^−1^ with a standard deviation of 0.59 kJ mol^−1^. Based on the calculated rate constant *k* (data from WinDNMR) at several temperatures (219–165K), the values Δ*G*_2_*^#^* for all measuring points were estimated (see Supporting Information Table S6). The obtained average value of 35.30 kJ mol^−1^ was very close to that determined using equation **b**. The difference between both Δ*G^#^* values reached 0.2 kJ.mol^−1^. The activation energy (*Ea*) was calculated by performing an *Arrhenius* plot (ln k vs. 1/T). The plot slope (*E_a_* = *R*.slope) allowed us to estimate the *E_a_* value (43.10 kJ mol^−1^). The corresponding enthalpy Δ*H^#^* (41.58 kJ mol^−1^) was calculated using the following equation: Δ*H^#^* = *E_a_ − *R*T_c_*. Bearing in mind the average Δ*G*_2_*^#^* value, the parameter Δ*S^#^* = 34.30 kJ mol^−1^ from equation Δ*S^#^* = (Δ*H^#^*) − Δ*G^#^*)/ *T_c_* was calculated. Additionally, from the slope of the Eyring plot (ln(*k*/T) vs. 1/T), the Δ*H*^#^ value (41.47 kJ mol^−1^) was estimated and the intercept of them allowed for the Δ*S^#^* (34.46 kJ mol^−1^.K) calculation. The calculated *Arrhenius* and *Eyring* plots Δ*H^#^* and Δ*S^#^* were similar with a difference of 0.11 kJ mol^−1^ and 0.16 kJ mol^−1^ K, respectively (Table 5).

**Figure 13:**
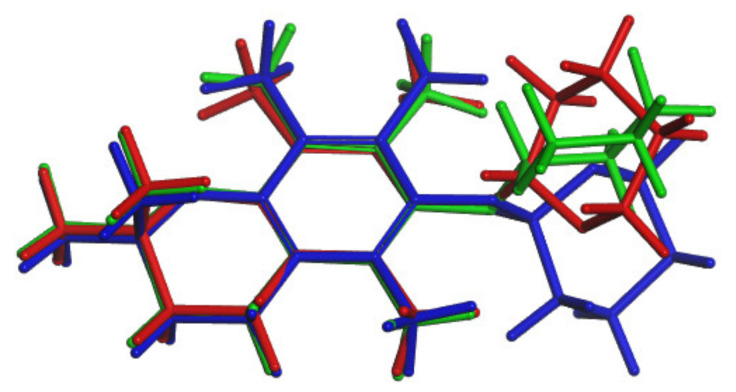
Conformations of **3**: a (**green**), TS (**red**), h (**blue**).

To study, at the molecular level, the mechanism of the **3a**–**3h** conformation conversion of the transition state (TS), calculations were conducted at the DFT level. The structure of TS can be found in Figure 13 and Figure S8. During this reaction, the conformation of tetrahydropyran did not change and remained in the form of a chair (Figure 13). The Δ*E^#^* and *E_a_* calculated values are presented in Table 6.

## 3. Materials and Methods

### 3.1. Synthesis

The 2,2,5,7,8-pentamethylchroman-6-ol (2) was obtained by acid-catalyzed condensation of trimethylhydroquinone with isoprene according to the method of Smith et al. [28]. The crude products were purified by medium pressure liquid chromatography (MPLC )chromatography, and resulted in a sample with an ^1^H NMR and ^13^C NMR spectra identical to those reported in the literature [29,30]. The ethers 4 and 5 were obtained from chroman-6-ol (2) following methylation [31,32] and sialylation by TBDMSCl while the THP ether 3 was obtained by the reaction of 2 in DCM sol. with 3,4-dihydro-2H-pyran in the presence of triphenylphosphine hydrogen bromide (10 mol%). The crude ethers 3–5 were purified by conducting MPLC on a Büchi Sepacore Easy Purification System, Büchi, India, and using cartridges packed with silica gel 230–400 mesh (Sigma-Aldrich) and an appropriate solvent system as an eluent. Melting points were measured on a Boëtius apparatus and were left uncorrected. The TLC was carried out on silica gel (60 GF 254, Merck). Detection was performed using UV-light followed by charring with sulfuric acid (10%) in ethanol. IR spectra were recorded on a Nicolet Magna 550 FTIR spectrometer, Thermo-Fisher, Waltham, MA, USA. Mass spectra were performed on an MS AMD-604 spectrometer.

### 3.2. NMR Analysis

The **^1^**H and **^13^**C NMR spectra were recorded on a Bruker Avance spectrometer operating at 400 and 100 MHz for ^1^H and ^13^C, respectively. Chemical shifts are reported in ppm (δ), downfield from tetramethylsilane (TMS). Splitting patterns are designated as follows: by = broad signal; s = singlet; d = doublet; t = triplet; dd = doublet of doublets, etc. *J*-couplings are given in Hz; *J*_HH_ were obtained in the first-order analysis. The spectra were assigned with the aid of 2D shift correlation NMR experiments (COSY, HSQC and HMBC). For routine (r.t) ^1^H and ^13^C NMR, the CDCl_3_ was used as a solvent. For variable-temperature measurement, deuterated dimethyl chloride (CD_2_Cl_2_) was used. The temperature was calibrated using methyl alcohol for temperatures below ambient.

Cross polarization magic angle spinning (CPMAS) solid-state ^13^C NMR was recorded on the Bruker MSL-300 instrument (Bruker, Billerica, MA, USA) at 75.5 MHz. The samples were spun at 10 kHz, with a contact time of 4 ms, a repetition time of 6 s and a spectral width of 20 k Hz for a total of 700–900 scans. Chemical shifts were calibrated indirectly through the glycine C=O signals recorded at 176.3 ppm relative to TMS. To identify the signals of strongly dipolar carbon coupled with hydrogen atoms in the NMR spectrum, ^13^C CPMAS Dipolar Dephase (DD) spectra were also recorded using the interrupted decoupling technique with a delay time of 50 µs. Dynamic ^13^C NMR simulations were performed using H. Reich WinDNMR free-shareware, Version 7.1.13, Hans J. Reich, Wisconsin, WI, USA [27].

### 3.3. X-ray Diffraction Measurements

Single crystals of 3(R/S), 4 and b were obtained by slow evaporation of the methanol solution at room temperature. Suitable crystals were selected, and X-ray data were collected on the Oxford Diffraction SuperNova DualSource diffractometer with the use of the monochromated Cu Kα X-ray source (λ = 1.54184). The crystals were kept at 100 K during data collection. Data reduction and analytical absorption correction were performed with CrysAlis PRO version 1.171.35.11; Agilent Technologies: Yarnton, Oxfordshire, UK [33]. Using Olex2 version. 1.3; OlexSys Ltd., Durham, UK, [34], all three structures were solved with the ShelXS [35] structure solution program using Direct Methods and refined with the ShelXL, version 6.14, Bruker Analytical X-ray Instruments. Madison, Wisconsin, WI, USA [35] refinement package using Least Squares minimization. A summary of relevant crystallographic data is provided in Table 1.

Hydrogen atoms were introduced in calculated positions with an idealized geometry and constrained using a rigid body model with isotropic displacement parameters equal to 1.2 or 1.5 of the equivalent displacement parameters of the parent atoms. The non-hydrogen atoms were refined anisotropically. The asymmetric unit of 3 contained 2 molecules (denoted as 3A and 3B). The tetrahydro-2H-pyranyl fragment and C8bB methyl group were disordered. The disorders were modelled in two conformations with parameter restraints (SIMU and ISOR instructions). The final check CIF/PLATON report signaled three alerts level. However, neither the O2 atom nor the O3 (O3”) atom act as a donor of the proton. The positions of disordered atoms of 3 were found on the electron density map.

The crystal structure of 5 (containing molecules 5A and 5B) was not disordered. The final check CIF/PLATON reported signals of one alert level B: PLAT097_ALERT_2_B Large Reported Max. (Positive) Residual Density was 1.42 eA-3. The residual electron density was not identified and the distance Si…Q-peak was equal to 1.830 Å.

CCDC-1815738 (3), CCDC-1815724 (4) and CCDC-1815726 (5) contain the supplementary crystallographic data for this paper. The data can be obtained free of charge from The Cambridge Crystallographic Data Centre via http://www.ccdc.cam.ac.uk/conts/retrieving.html accessed on 1 June 2022.

### 3.4. DFT Calculations for Isolated Molecules

The DFT calculations of geometry optimization and NMR shielding constants for the isolated molecules of 3 were carried out with the Gaussian 16 program [36]. The molecules from the Conformers search (as described in the “Conformers calculations” section) were taken as the starting geometries to optimize all atom’s positions. All electron computations were completed by employing the 6-311++G(d,p) basis set and B3LYP functional, with Grimme’s D3 dispersion force corrections (B3LYP-D3). The polarizable continuum model (PCM) [37] was used for implicit solvation, which dichloromethane chosen as the solvent (dielectric constant equals ε = 8.93).

The natural mode frequencies were calculated in harmonic approximation to confirm that each structure was not in a transition state. The existence of only positive frequencies confirmed the nature of the stationary points on the potential surface.

The resulting geometries were used for the calculation of NMR shielding constants using the GIAO approach. To compare the theoretical and experimental data, the calculated chemical shielding constants (σiso) were converted to chemical shifts (δt) using the following equation: δt = σTMS—σiso, where σTMS stands for the shielding constant of the tetramethylsilane (TMS) carbon atom calculated at the same level of theory.

To obtain the activation energy of the transition state, the structure of the transition state was searched at the same level of theory (B3LYP-D3, 6-311++G(d,p)) using the QST2 method, which utilizes the optimized structures of reactants and products. The transition state structure obtained from the QST2 calculations was then optimized by performing TS optimization and frequency calculations. The resulting structure was confirmed to be a transition state as one imaginary frequency was found.

### 3.5. Periodic DFT Calculations for 3

The density functional theory (DFT) calculations of geometry optimization and NMR shielding constants were carried out using the CASTEP program [38] implemented in the Materials Studio 2017 software, BIOVIA, San Diego, CA, USA, using the plane-wave pseudopotential formalism and the Perdew–Burke–Ernzerhof (PBE) exchange-correlation functional [39] defined within the generalized gradient approximation (GGA) using the Grimme method for dispersion correction (DFT-D) [40].

The calculations were performed using ultrasoft pseudopotentials calculated on the fly. Geometry optimization was carried out using the Broyden–Fletcher–Goldfarb–Shanno (BFGS) [41] optimization scheme and smart method for finite basis set correction. The quality of calculations was set to ultra-fine as implemented in the CASTEP standards. The convergence criteria were set at 5 × 10^−6^ eV/atom for the energy, 1 × 10^−2^ eV/Å for the interatomic forces, 2 × 10^−2^ GPa for the stresses and 5 × 10^−4^ Å for the displacements. The fixed basis set quality method for the cell optimization calculations and the 5 × 10^−7^ eV/atom tolerance for SCF were used. The kinetic energy cutoff for the plane waves was set to 630 eV. Brillouin zone integration was performed using a discrete 3 × 3 × 1 Monkhorst-Pack k-point sampling for a primitive cell.

The optimized structures were then used for the NMR parameter calculations using the Gauge Including Projector Augmented Wave (GIPAW) method of Pickard and Mauri [42]. To facilitate the comparison of the theoretical and experimental data, the calculated shielding constants (σiso) were converted into chemical shifts (δt), using the following equation: δt = (σGly + δGly) − σiso, where σGly and δGly stand for the calculated shielding constant and the experimental chemical shift, respectively, of the glycine carbonyl carbon atom (176.3 ppm).

### 3.6. Conformers Calculations

The conformational search of the studied molecules was performed using the Conformers module of Materials Studio 2017, by applying the Metropolis Monte Carlo algorithm based Boltzmann jump search method. A total number of 26 conformers of 3 were generated (named a-z), applying 500 perturbations per jump within a torsion angle window adjusted to accept 50% of the generated conformers. Geometry optimization was performed on each conformer at the molecular mechanics level by the provided Smart algorithm (COMPASS force field, ultra-fine quality and maximum 5 × 104 iterations). Then, the conformations were used as initial structures for the Gaussian DFT calculations.

## 4. Conclusions

This work was a continuation of the research on 2,2,5,7,8-pentamethylchroman-6-oles with different substituents at the C6 position. The analysis was performed through experimental (NMR and X-ray) as well as theoretical (DFT) techniques. The crystallographic and NMR data obtained here are an important contribution to the understanding of stereo-electron effects occurring in 2,2,5,7,8-pentamethylchroman-6-oles—the main element of the biologically active structure of α-tocopherol (Vitamin E).

The following progress was made a result of the work carried out:

- For the first time, 2,2,5,7,8-pentamethylchroman-6-oles: methyl (4), TBDMS (**5**) and THP (**3**) ether XRD structures were described;
- The effects of the introduction of methyl, TBDMS and THP residue at the C6 position in 2,2,5,7,8-pentamethylchroman-6-ol were investigated. The deformation in the symmetry of the aromatic ring and its deviation from planarity compared to the substituent phenol were analyzed. A shortening of the C8a-O1 bond (c.a. 0.3 Å) with the simultaneous lengthening of the C6-C7, C8-C8a, C8a-C4a and C4a-C5 bonds in all analyzed ethers **3–5** were observed, as well as a significant deviation from the plane in the aromatic ring (up to 5–6o from planarity);
- In the case of THP ether (**3**), based on the signals C7 and C5 full shape line analysis (^13^C DNMR), the temperature of coalescence was estimated (at 183K ± 5°). Using WinDNMR software and the Arrhenius and Eyring equations, the kinetic parameters for ether **3** were calculated. The calculated *ΔG^#^* barrier rotation value (35.10 kJ mol^−1^ ± 0.59) was half of that previously determined for α-tocopherol or chroman-6-ol esters (c.a 59–63 kJ mol^−1^);
- The GIPAW DFT NMR calculations facilitated the unambiguous assignment of the signals in the ^13^C CP MAS spectra of (**3**);
- DFT calculations helped to explain the splitting of some signals in the variable temperature ^13^C NMR spectra of (**3**) by optimization and NMR property calculations of the structures were found during the conformational search.

## Figures and Tables

**Table 1:** Crystal data and structure refinement for ethers **3**(*R/S*), **4** and **5**.

Identification Code	3(*R*/*S*)	4	5
Empirical formula	C_19_H_28_O_3_	C_15_H_22_O_2_	C_20_H_34_O_2_Si
Formula weight	304.41	234.32	334.56
Crystal size/mm^3^	0.284 × 0.242 × 0.115	0.468 × 0.269 × 0.068	0.395 × 0.16 × 0.041
Crystal system	monoclinic	monoclinic	monoclinic
Space group	I2/a	P2_1_/n	P2_1_/c
a/Å	26.978 (6)	12.960 (5)	21.564 (9)
b/Å	10.423 (2)	5.626 (7)	8.172 (5)
c/Å	39.524 (2)	18.066 (9)	22.833 (4)
α/°	90	90	90
β/°	142.58 (8)	95.09 (3)	95.33 (3)
γ/°	90	90	90
Volume/Å^3^	6752.3 (13)	1312.3 (3)	4006.6 (8)
Z	16	4	8
ρ_calc_g/cm^3^	1.198	1.186	1.109
μ/mm^−1^	0.625	0.601	1.078
2Θ range for data collection/°	6.662 to 152.732	8.066 to 152.906	7.778 to 149.008
Reflections collected	28329	25650	42438
Independent reflections	7073 [R_int_ = 0.0238, R_sigma_ = 0.0175]	2745 [R_int_ = 0.0527, R_sigma_ = 0.0222]	8196 [R_int_ = 0.0293, R_sigma_ = 0.0197]
Data/parameters/restraints	7073/535/48	2745/160/0	8196/435/0
Goodness-of-fit on F^2^	1.103	1.055	1.038
Final R indexes [I > =2σ (I)]	R_1_ = 0.0473, wR_2_ = 0.1220	R_1_ = 0.0642, wR_2_ = 0.1775	R_1_ = 0.0487, wR_2_ = 0.1391
Final R indexes [all data]	R_1_ = 0.0495, wR_2_ = 0.1238	R_1_ = 0.0697, wR_2_ = 0.1877	R_1_ = 0.0535, wR_2_ = 0.1474
Largest diff. peak/hole /eÅ^−3^	0.26/−0.23	0.30/−0.42	1.42/−0.36

**Table 2:** Experimental CP MAS (Exp.) and calculated (GIPAW) ^13^C NMR chemical shifts (δ, ppm) for ether **3**.

Atom Number	Exp.	GIPAW	Δ(Exp.-GIPAW)
Molecule A			
2	73.4	77.66	−4.26
2a	25.6	23.56	2.04
2b	29.8	27.17	2.63
3	31.1	28.12	2.98
4	21.9	20.41	1.49
4a	118.3	120.05	−1.75
5	129.7	132.14	−2.44
5a	14.4	12.95	1.45
6	146.4	149.71	−3.31
7	126.4	129.35	−2.95
7a	14.4	13.01	1.39
8	121.4	123.11	−1.71
8a	148.4	150.95	−2.55
8b	11.8	9.58	2.22
1′	103.7	108.71	−5.01
2′	32.3	29.52	2.78
3′	24.1	22.64	1.46
4′	24.1	23.46	0.64
5′	67.1	67.24	−0.14
Molecule B			
2	73.4	77.74	−4.34
2a	25.6	23.96	1.64
2b	31.1	28.98	2.12
3	32.3	29.71	2.59
4	21.9	20.75	1.15
4a	118.3	119.79	−1.49
5	129.7	133.2	−3.5
5a	12.8	11.53	1.27
6	146.4	149.46	−3.06
7	126.4	129.77	−3.37
7a	14.4	13	1.4
8	121.4	123.12	−1.72
8a	148.4	151.28	−2.88
8b	11.8	8.71	3.09
1′	103.7	108.34	−4.64
2′	32.3	30.75	1.55
3′	24.1	23.45	0.65
4′	21.9	21.95	−0.05
5′	70	69.53	0.47

**Table 3:** The experimental solution (Exp.) and calculated (GIAO) ^13^C NMR chemical shift values for ether **3** [ppm]. Energy [kJ/mol] is the electronic energy of the conformation.

Conformation Symbol		a	h	a−h
Energy [kJ/mol]		−2,513,941.28	−2,513,915.03	−26.25
Atom Number	Exp.	GIAO		
2	72.6	73.65	73.95	−0.3
2a	26.9	25.85	25.83	0.02
2b	26.8	27.43	27.47	−0.04
3	32.9	30.8	30.8	0
4	21.1	19.66	19.83	−0.17
4a	117	118.48	117.25	1.23
5	126.4	127.05	124.82	2.23
5a	11.9	9.52	9.15	0.37
6	147.3	148.38	149.06	−0.68
7	128.2	129.42	130.89	−1.47
7a	13.8	13.35	12.87	0.48
8	122.6	124.46	125.5	−1.04
8b	12.9	12.45	12.1	0.35
8a	148	149.75	149.47	0.28
1′	103.7	104.95	103.53	1.42
2′	31.3	30.67	31.06	−0.39
3′	21.2	21.5	21.3	0.2
4′	25.2	24.11	23.88	0.23
5′	65.1	65.45	65.87	−0.42

**Table 4:** The calculated Δ*G*^#^ values for ether **3**.

Atom	Max ∆ν * [Hz]	*T*c [K]	Δ*G^#^* ** [kJ mol^−1^]	*k*_exch_ [Hz]
C7	153	183	34.77	339.7
C5	161		35.12	357.4
C8	75	181	35.87	166.5
C4a	74.5		35.88	165.4
C7a	24	166	34.35	53.3
C5a	20		34.60	44.4

Average **Δ*G^#^*** = 35.10 kJ mol^−1^ (standard dev. = 0.59). * from spectra recorded at 165K, ** values determined using the **b** equations.

**Table 5:** Calculated kinetic parameters for ether **3**—standard deviations in parenthesis.

Δ*G*_1_*^#^* [kJ mol^−1^]	Δ*G*_2_*^#^* [kJ mol^−1^]	*E_a_* [kJ mol^−1^]	Δ*H*_1_*^#^*[kJ mol^−1^]	Δ*S^#^* [kJ mol^−1^ K]
35.10 (0.59) ^a^	35.30 (1.76) ^b^	43.10 ^c^	41.58 ^c^/41.47^d^	34.30 ^c^/34.46 ^d^

a/-the value found at *Tc* with Equation (2). b/-the average value from WinDNMR simulation. c/-values determined using the Arrhenius plots. d/-data determined using the Eyring plots.

**Table 6:** Calculated (DFT) electronic energetic parameters for ether **3**.

Conformation Symbol	a	h	*TS*	Δ*E^#^* (h-a)	*E_a_* (TS-a)
Energy [kJ/mol]	−2,513,941.28	−2,513,915.03	−2,513,910.39	26.25	30.89

## Data Availability

To access the data supporting the reported results, please contact the corresponding author (P.W. or Ł.S.)

## Supplementary Materials

The following supporting information can be downloaded at: https://www.mdpi.com/article/10.3390/molecules27144630/s1, Figure S1. Crystal packing scheme in 3. A view of the unit cell in the [010] direction. Hydrogen atoms are omitted for clarity; Figure S2. The dimeric structure formed in crystals of **4** a) and supramolecular structure formed by methyl ether **4** (view of the unit cell in the [010] direction); Figure S3. Crystal packing scheme in **5** with torsion angle *θ*[°] (red) and *γ*[°] (blue) values. A view of the unit cell in the [010] direction. Hydrogen atoms are omitted for clarity; Figure S4. The short contact in the crystal structure of **5**; Figure S5 Dynamic ^13^C NMR spectra of **4**: (a) 298.3, (b) 239.6, (c) 210.2, (d) 178.4 K; Figure S6 Dynamic ^13^C NMR spectra of 5 (ether TBDMS): (a) 298, (b) 250, (c) 200 and (d) 190 K; Figure S7 Dynamic ^13^C NMR of **3** in differentia temperature of measurement (temp. range 290–165 K); Figure S8 Conformations of **3**: a (green), TS (red), h (blue); Table S1. Selected bond lengths [Å] in ethers 3, 4 and 5 determined by XRD data; Table S2. Selected bond angles [°] in ethers 3, 4 and 5 determined by XRD data; Table S3. Selected torsion angles [°] in ethers 3, 4 and 5 determined by XRD data; Table S4. The bond length comparison in ethers **4**, **3**A-B and **5**A-B and differences in bond length between **4** and ethers **3** and **5** with 3σ values); Table S5. The bonds length comparison between 4-methoxy-2,3,5,6-tetramethylphenol (4MTP, CCDC MOPHLA) and ethers **4**, **3**A-B and **5**A-B; Table S6. Data for ether **3** from WinNMR; Table S7. Energies [kJ/mol], relative energies [kJ/mol] and calculated NMR isotropic chemical shift values [ppm] of the studied conformations of **3**.

## Sample Availability

Samples of the compounds **3**–**5** are available from the authors (P.W.).

## Abbreviations

1	d-α-tocopherol
2	chroman-6-ol
2a	Trolox C
3	2,2,5,7,8-pentamethyl-6-((tetrahydro-2H-pyran-2-yl)oxy) chroman
4	6-methoxy-2,2,5,7,8-pentamethylchromane
5	tert-butyldimethyl((2,2,5,7,8-pentamethylchroman-6-yl)oxy)silane
CCDC	Cambridge Crystallographic Data Centre
CP	cross polarization
DD	dipolar diphase
DNMR	dynamic nuclear magnetic resonance
DFT	density functional theory
DQF-COSY	double quantum filter correlation spectroscopy
Ea	energy of activation
ECD	electronic circular dichroism
Δ*G^#^*	free enthalpy of activation
GGA	generalized gradient approximation
GIPAW	Gauge Including Projector Augmented Wave
Δ*H^#^*	enthalpy of activation
HMBC	heteronuclear multiple bond correlation
HSQC	heteronuclear single quantum coherence
MAS	magic angle spinning
MPLC	medium pressure liquid chromatography
NMR	nuclear magnetic resonance
PBE	Perdew–Burke–Ernzerhof exchange-correlation functional
PCM	polarizable continuum model (implicit solvation scheme)
Δ*S^#^*	entropy of activation
SCXRD	single crystal X-ray diffraction
TBDMS	tert-butyldimethylsilyl
Tc	coalescence temperature
THP	tetrahydropyran
TMS	tetramethylsilane
TS	transition state between the a and h conformations of 3
