# Supplementary material for: 13C CP MAS NMR and DFT Studies of 6-Chromanyl Ethereal Derivatives
Source: Molecules. 2022 Jul 20;27(14):4630. doi: 10.3390/molecules27144630 (PMC9323032; doi:10.3390/molecules27144630)
Supplement: Supplementary file 1 [file molecules-27-04630-s001.zip › molecules-1829311-supplementary.pdf]

Electronic supplementary information

for

# $^{13}$C CP MAS NMR and DFT studies of 6-chromanyl etheral derivatives.†

Piotr Wałejko[1*], Łukasz Szeleszczuk[2*], Dariusz Maciej Pisklak[2], and Sławomir Wojtulewski[1]

[1]University of Białystok, Faculty of Chemistry, Ciołkowskiego 1K, 15-245 Białystok, Poland

[2] Department of Physical Chemistry, Chair of Physical Pharmacy and Bioanalysis, Faculty of Pharmacy, Medical University of Warsaw, Banacha 1 Str., 02-093 Warsaw, Poland

* Correspondence: lukasz.szeleszczuk@wum.edu.pl; Tel.: +48-501-255-121 (Ł.Sz.) ; tel: +48 85 7388086; pwalejko@uwb.edu.pl (P.W.)

† Dedicated to Prof. Stanisław Witkowski on the occasion of his 70th birthday.

## Table of contents

**Experimental section**

1. Synthesis and spectroscopic data of ethers **3-5**

**Fig. S1**. Crystal packing scheme in **3**. A view of the unit cell in the [010] direction. Hydrogen atoms are omitted for clarity.

**Fig. S2**. The dimeric structure formed in crystals of **4** a) and supramolecular structure formed by methyl ether **4** (view of the unit cell in the [010] direction).

**Fig. S3**. Crystal packing scheme in **5** with torsion angle $\theta$[°] (red) and $\gamma$[°] (blue) values. A view of the unit cell in the [010] direction. Hydrogen atoms are omitted for clarity.

**Fig. S4**. The short contact in crystal structure of **5**.

**Fig. S5** Dynamic $^{13}$C NMR spectra of **4**: a) 298.3, b) 239.6, c) 210.2, d) 178.4 K.

**Fig. S6** Dynamic $^{13}$C NMR spectra of **5** (eter TBDMS): a) 298, b) 250, c) 200 and d) 190 K.

**Fig. S7** Dynamic $^{13}$C NMR of **3** in differentia temperature of measurement (temp. range 290 – 165 K).

**Fig. S8** Conformations of **3**: a (green), TS (red), h (blue).

**Table S1**. The bonds length comparison in ethers **4**, **3**A-B and **5**A-B and differences in bond lengthens between **4** and ethers **3** and **5** with 3σ values).

**Table S2**. The bonds length comparison between 4-methoxy-2,3,5,6-tetramethylphenol (4MTP, CCDC MOPHLA) and ethers **4**, **3**A-B and **5**A-B.

**Tabela S3**. Data for ether **3** from WinNMR.

Table S4. Energies [kJ/mol], relative energies [kJ/mol] and calculated NMR isotropic chemical shift values [ppm] of the studied conformations of **3**.

1. Synthesis and spectroscopic data of ethers **3-5**

2,2,5,7,8-pentamethyl-6-((tetrahydro-2*H*-pyran-2-yl)oxy)chromane (**3**).

The chroman-6-ol (**2**; 1 equiv.) was stirred overnight at room temperature with 3,4-dihydro-2H-pyran (3 equiv.) in diethyl ether solutions containing catalytic amount of PTSA. In next step the reaction mixture was incubated with excess of solid $K_2CO_3$ and after aqueous work-up the resulted organic fraction was concentrated in vacuum to dryness. The crude ether **3** was purified by MPLC (hexane-ethyl acetate. 15:1 v/v)). Re-crystallization from ethanol gave the desired product **3** with 60% yield.

$^{1}$H NMR (δ, 400 MHz, $CDCl_3$): 4.67 (dd, 1H, H-1′, $^{3}J_{H\text{-}H}$ = 6.9 Hz and 2.2 Hz), 4.09 (m, 1H, H-5′$_{ax}$), 3.48 (m, 1H, H-5′$_{eq}$), 2.62 (m, 2H, H-4), 2.21 (s, 3H, H-7a), 2.18 (s, 3H, H8b), 2.11 (s, 3H, H-5a), 1.95 (m, 2H, H-2′$_{ax}$ and 3′$_{ax}$), 1.87 (m, 1H, H-2′$_{eq}$), 1.81 (~t, 2H, H-3, $^{3}J_{H\text{-}H}$ = 7.1 Hz), 1.599 (m, 2H, H-4′ and 3′$_{eq}$), 1.315 (s, 6H, H-2a and 2b) ppm.

$^{13}$C NMR (δ, 150 MHz, $CDCl_3$): 148.00 (C8a), 147.33 (C6), 128.25 (C7), 126.38 (C5), 122.57 (C8), 117.04 (C4a), 103.73 (C1′), 72.64 (C2), 65.06 (C5′), 32.94 (C3), 31.29 (C2′), 26.86 (C2a), 26.80 (C2b), 25.24 (C4′), 21.16 (C3′), 21.06 (C4), 13.81 (C7a), 12.93 (C5a), 11.86 (C8b) ppm.

IR ($CHCl_3$) $\nu_{max}$/cm$^{-1}$: 3003,. 2977, 2945, 2855, 1456, 1412, 1382, 1370, 1264, 1167, 1125, 1102, 1077, 1035, 952, 926, 909, 897.

TOF MS ES$^+$: 327 [M + Na]$^+$; HRMS: m/z: calc. for $C_{19}H_{28}O_3Na$ [M + Na]$^+$: 327.1931; found: 327.1938

6-methoxy-2.2.5.7.8-pentamethylchromane (**4**)

The chroman-2-ol (**2**. 1 equiv.) and dimethyl sulfate (excess) were heating with stirring in dry suspension of $K_2CO_3$ in acetone. The cooled solution was poured into water and extracted with diethyl ether (3 × 50 mL). The combined organic extracts were washed with sodium bicarbonate, dried over anhydrous $Na_2SO_4$ and concentrated under reduced pressure. The residue was purified by flash column chromatography (hexane/ethyl acetate, 20:1, *v/v*) to afford ester **4** as a white solid with 70% yield.

The $^1H$ and $^{13}C$ NMR spectra were with good similarity to those described in literature. [Byzova. V. N.; Zakharova. E. I.; Zhukova. E. E.; Sarycheva. I. K.; Evstigneeva. R. P.; Journal of Organic Chemistry USSR (English Translation); vol. 24; nb. 11; (1988); p. 2135 - 2140; Zhurnal Organicheskoi Khimii; vol. 24; nb. 11; (1988); p. 2368 - 2374. and Al-Khayat. Isam; Dean. Francis M.; France. Steven N.; Matkin. David A.; Orabi. Mohamed O. A.; et al.; Journal of the Chemical Society. Perkin Transactions 1: Organic and Bio-Organic Chemistry (1972-1999); (1985); p. 1301 – 1310]

$^1H$ NMR (δ. 400 MHz. $CDCl_3$): 3.64 (s, 1H, H-1′), 2.63 (t, 2H, H-4, $^3J_{H\text{-}H}$ = 6.8 Hz), 2.20 (s, 3H, H-7a), 2.16 (s, 3H, H8b), 2.10 (s, 3H, H-5a), 1.82 (t, 2H, H-3, $^3J_{H\text{-}H}$ = 6.8 Hz), 1.33 (s, 6H H-2a and 2b) ppm.

$^{13}C$ NMR (δ, 150 MHz, $CDCl_3$): 147.88 (C8a), 149.45 (C6), 127.72 (C7), 125.70 (C5), 122.85 (C8), 117.31 (C4a), 72.71 (C2), 60.35 (C1′), 32.91 (C3), 26.8179 (C2a and 2b), 20.94 (C4), 12.52 (C7a), 11.74 (C8b), 11.66 (C5a) ppm.

TOF MS ES$^+$: 257 [M + Na]$^+$; HRMS: m/z: calc. for $C_{15}H_{22}O_2Na$ [M + Na]$^+$: 257.1512; found: 257.1519

*tert*-butyl-*di*-methyl((2.2.5.7.8-pentamethylchroman-6-yl)oxy)silane (**5**)

Trimethylamine (1.5 equiv.) under argon atmosphere was added to the chroman-2-ol (**2**, 1 equiv.) in dry THF solution and after 5 min. of stirring the *tert*-butyldimethylsilyl chloride (TBDMSCl, 1.1 equiv.) was added dropwise. The reaction after overnight stirring at room temperature was poured into water and extracted with diethyl ether (3 × 50 mL). The combined organic extracts were washed with sodium bicarbonate, dried over anhydrous $Na_2SO_4$ and concentrated under reduced pressure. The residue was purified by flash column chromatography (hexane/ethyl acetate, 30:1. v/v) to afford ester **5** with 80% yield.

$^1$H NMR (δ. 400 MHz. $CDCl_3$): 2.58 (t, 2H, H-4, $^3J_{H-H}$ = 6.8 Hz), 2.11 (s, 3H, H-7a), 2.09 (s, 6H, H-8b), 2.07 (s, 3H, H-5a), 1.80 (t, 2H, H-3, $^3J_{H-H}$ = 6.8 Hz), 1.30 (s, 6H, H-2a and 2b), 1.06 (s, 9H., H-2′), 0.13 (s, 6H, H-1′) ppm.

$^{13}$C NMR (δ, 150 MHz, $CDCl_3$): 146.05 (C8a), 144.14 (C6), 125.84 (C7), 123.50 (C5), 122.63 (C8), 117.25 (C4a), 72.45 (C2), 33.10 (C3), 26.77 (C2a and 2b) 26.12 (C2″), 21.21 (C4), 18.61 (C2′), 14.33 (C7a), 13.41 (C5a), 11.93 (C8b), -3.21 (C1′) ppm.

TOF MS ES$^+$: 357 [M + Na]$^+$; HRMS: m/z: calc. for $C_{20}H_{34}NaO_2Si$ [M + Na]$^+$: 357.2220; found: 357.2226

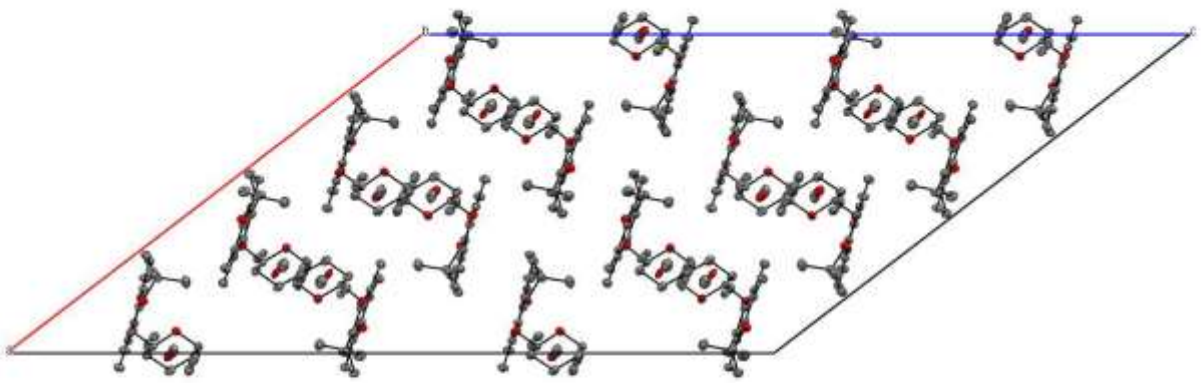

**Fig. S1**. Crystal packing scheme in **3**. A view of the unit cell in the [010] direction. Hydrogen atoms are omitted for clarity.

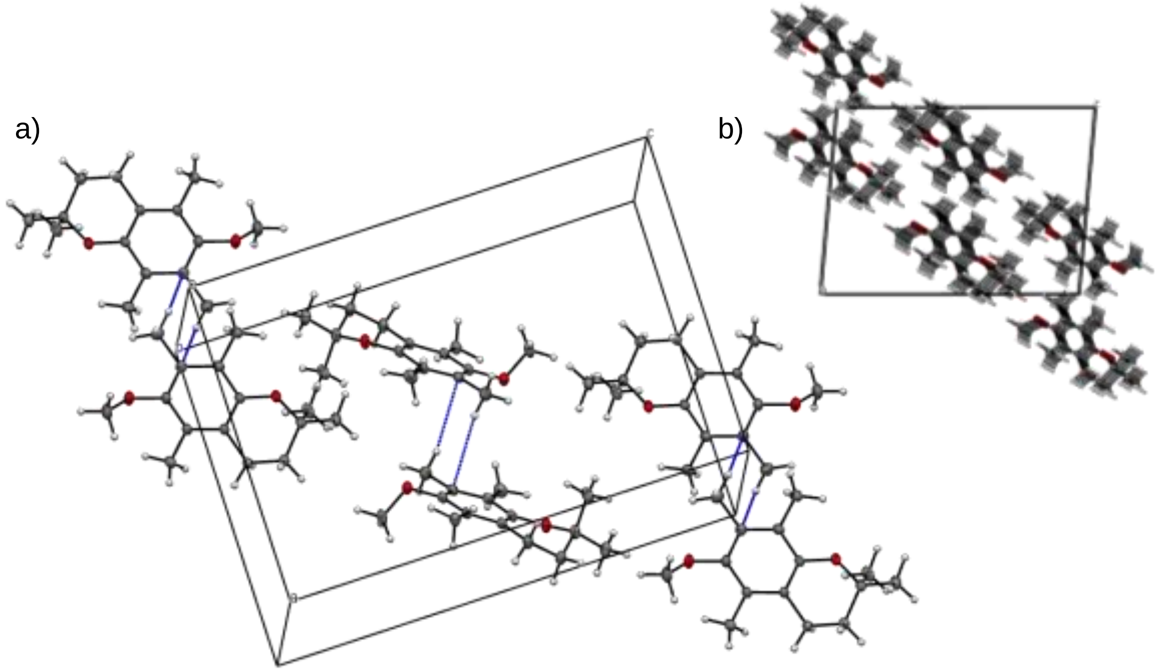


**Fig. S2**. The dimeric structure formed in crystals of **4** a) and supramolecular structure formed by methyl ether **4** (view of the unit cell in the [010] direction).

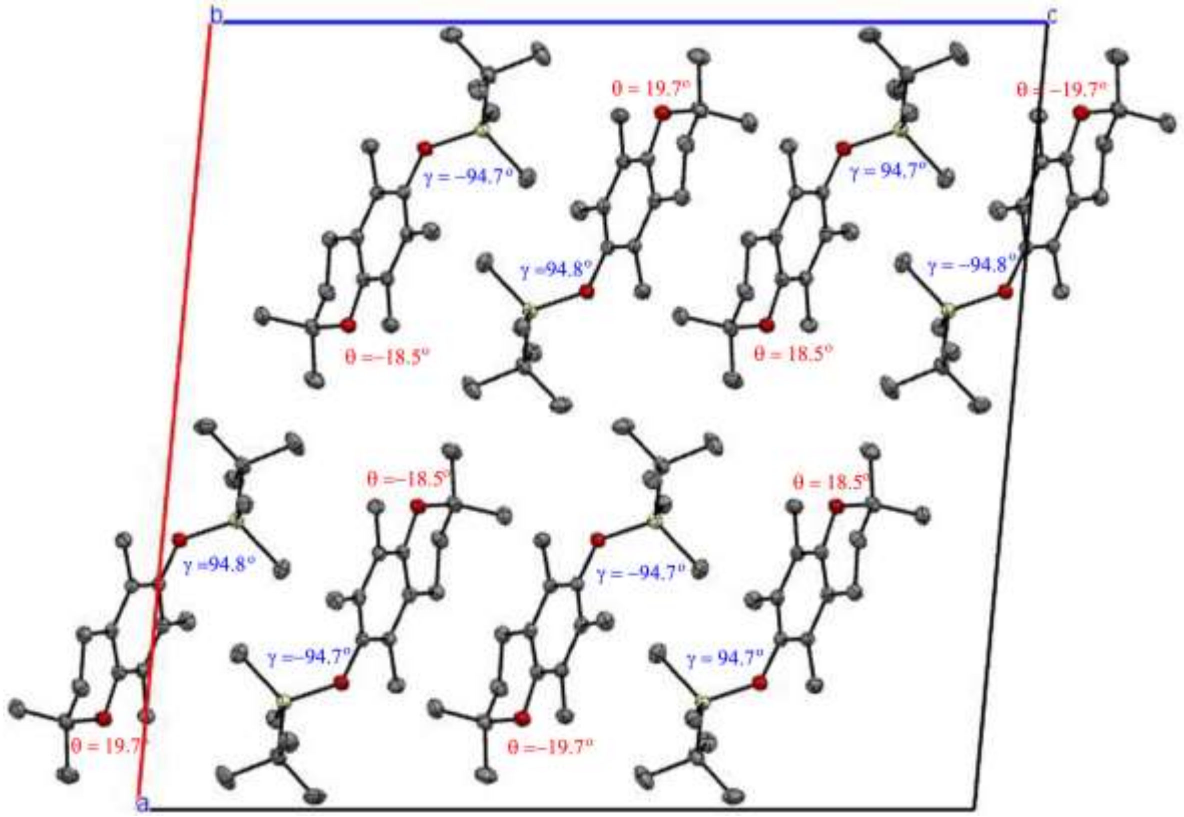


**Fig. S3**. Crystal packing scheme in **5** with torsion angle $\theta$[°] (red) and $\gamma$[°] (blue) values. A view of the unit cell in the [010] direction. Hydrogen atoms are omitted for clarity.

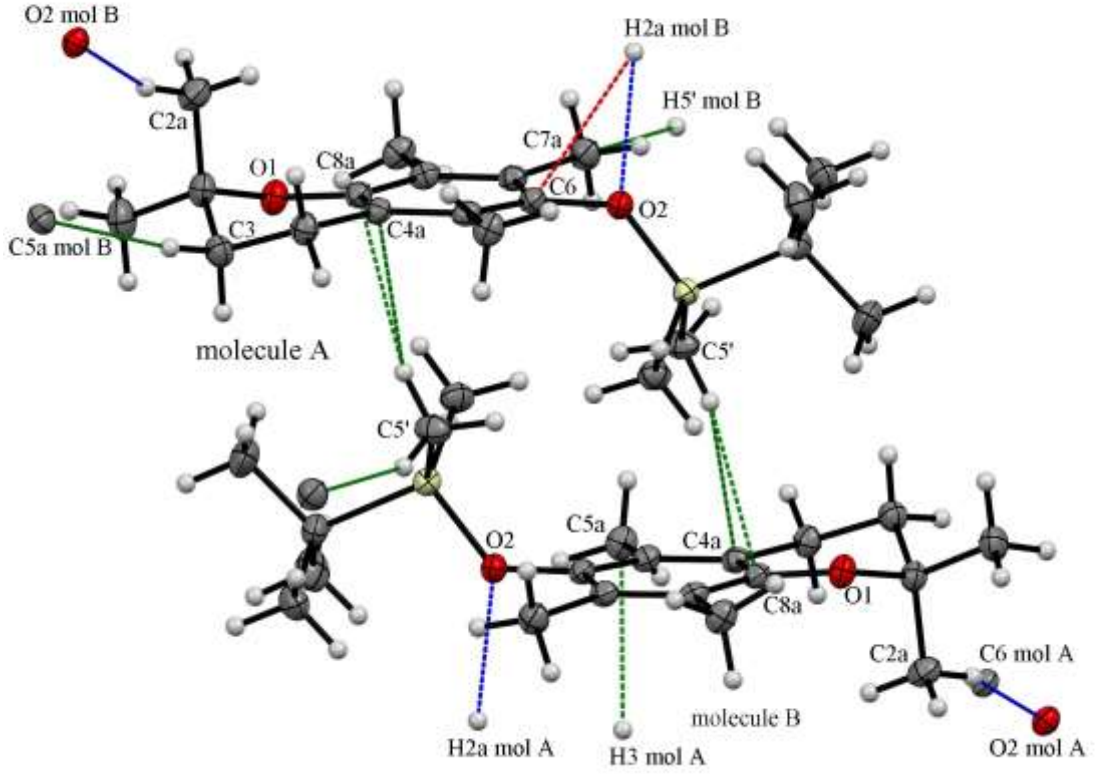


**Fig. S4**. The short contact in crystal structure of **5**.

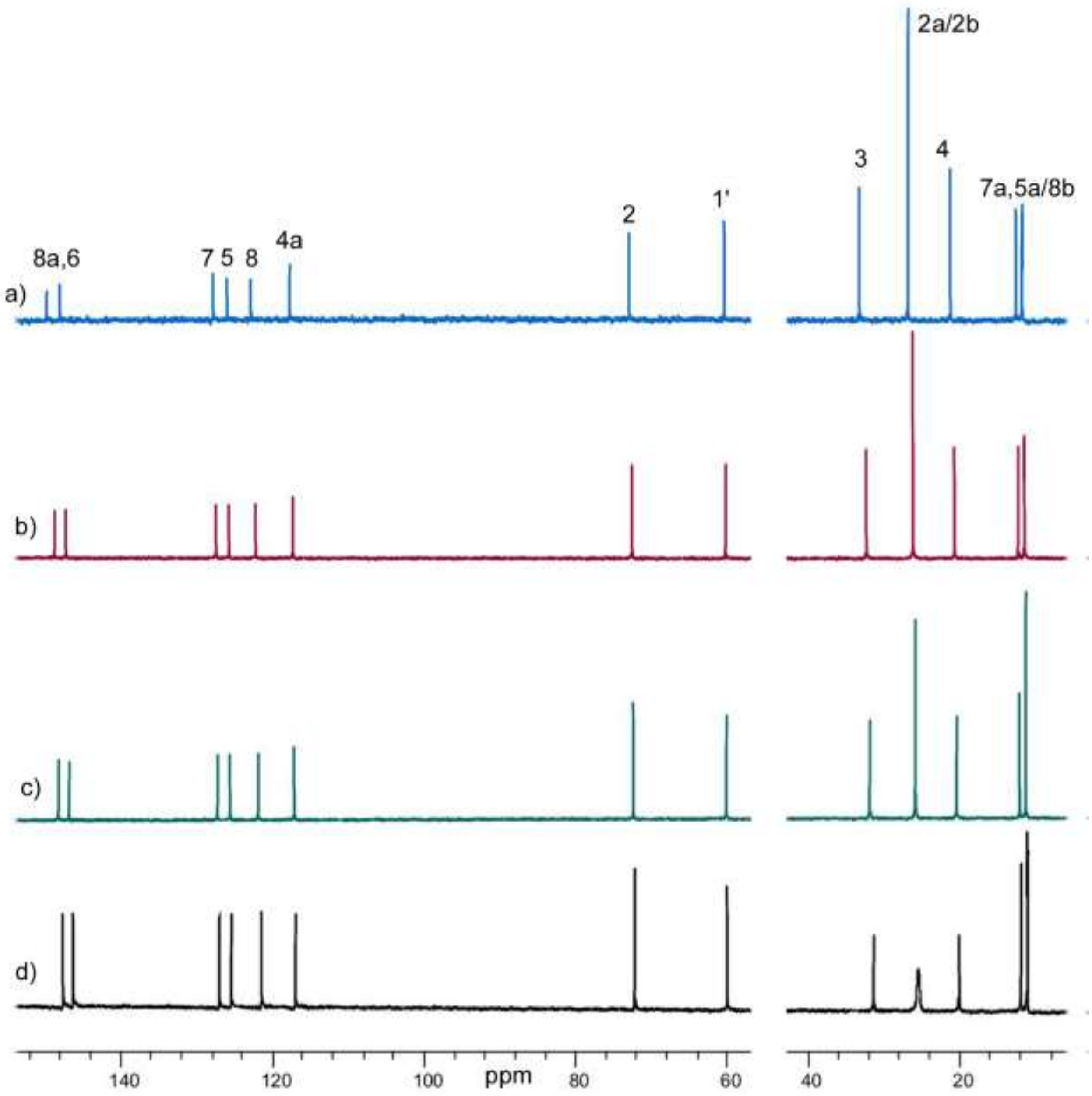

**Fig. S5** Dynamic $^{13}$C NMR spectra of **4**: **a**) 298.3, **b**) 239.6, **c**) 210.2, **d**) 178.4 K.

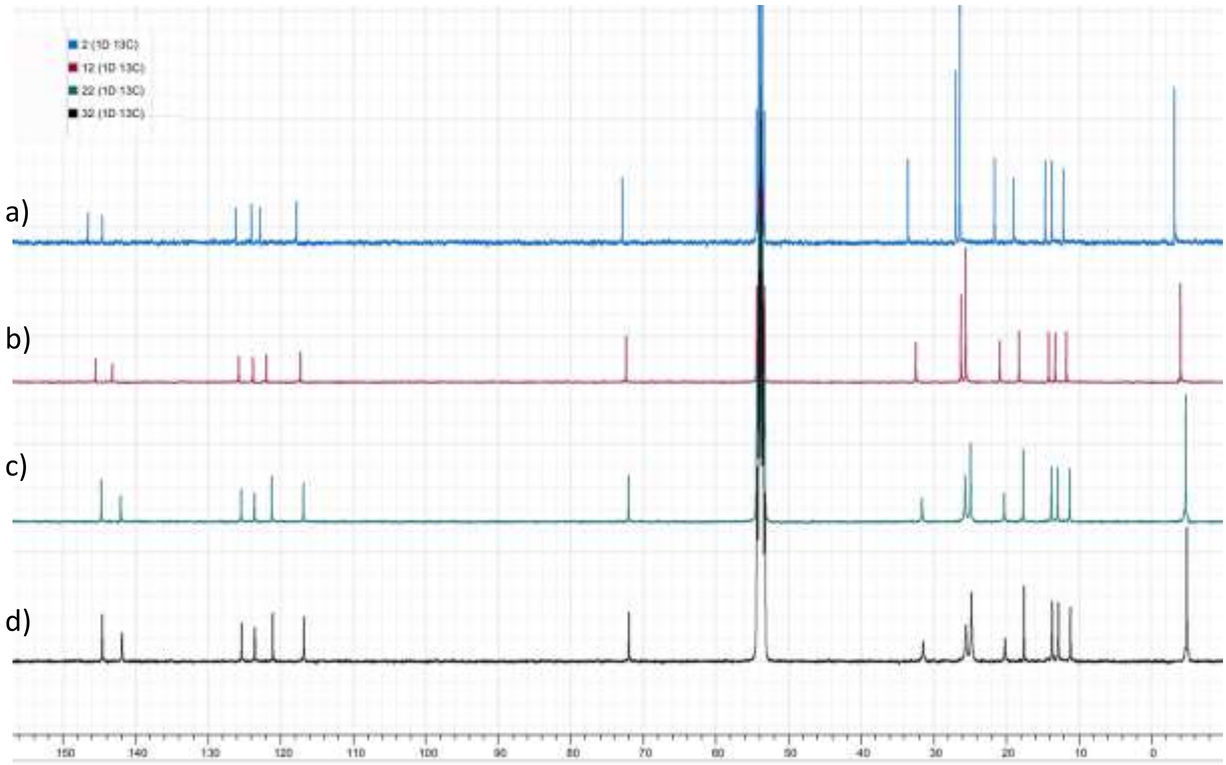


**Fig. S6** Dynamic $^{13}$C NMR spectra of **5** (eter TBDMS): **a**) 298, **b**) 250, **c**) 200 and **d**) 190 K.

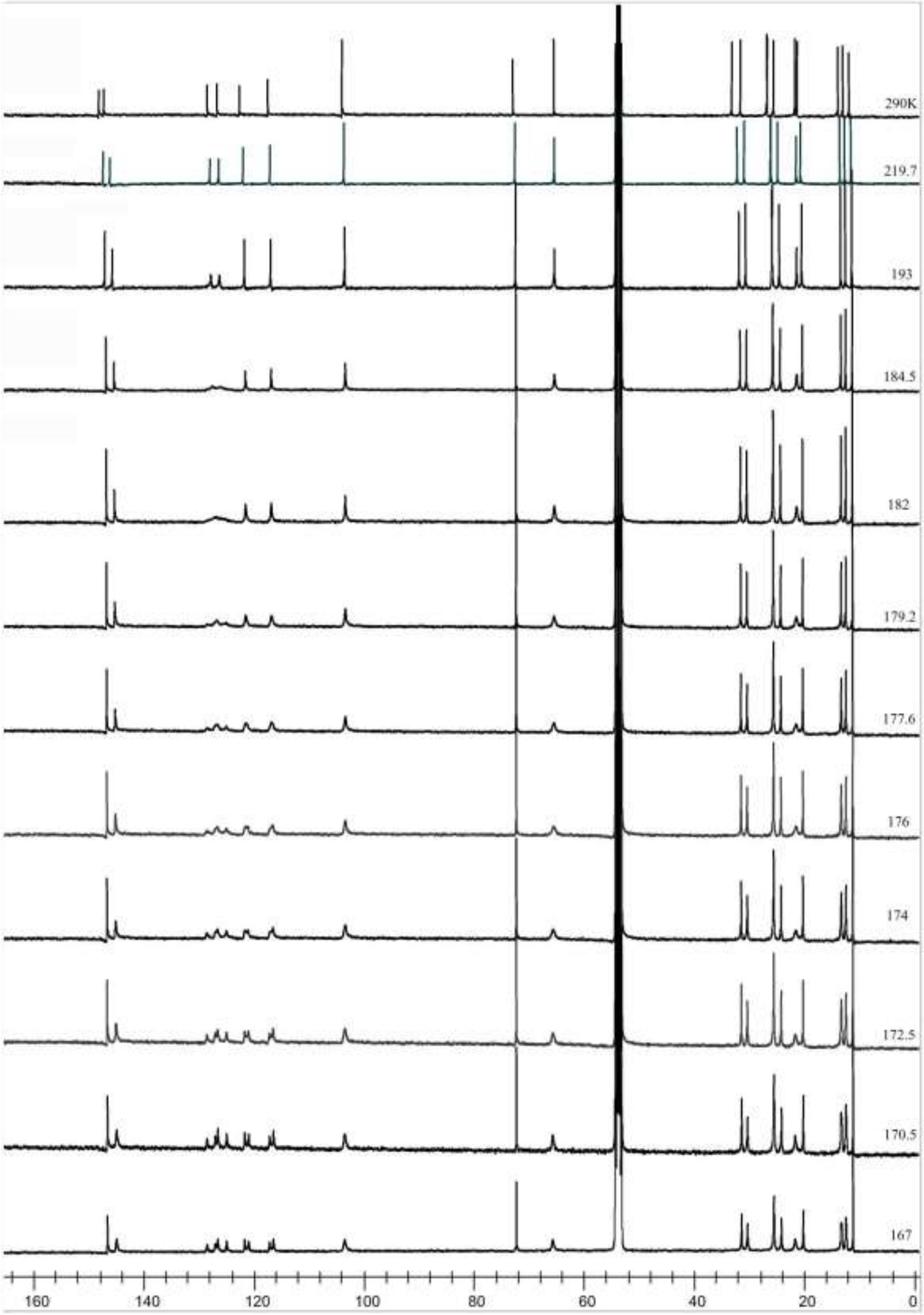


**Fig. S7** Dynamic $^{13}$C NMR of **3** in differentia temperature of measurement (temp. range 290 – 165 K).

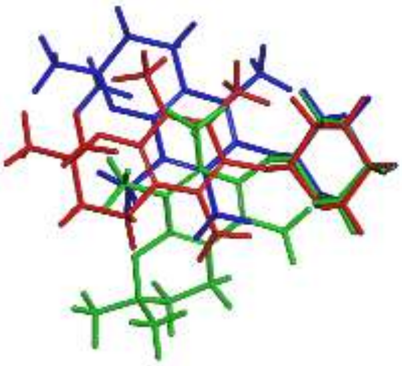

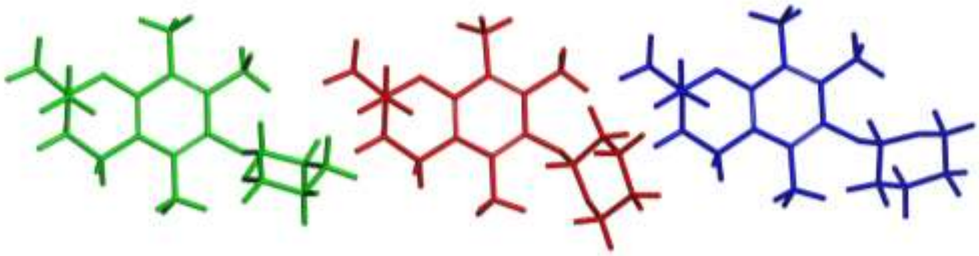

**Fig. S8** Conformations of **3**: a (green), TS (red), h (blue).

**Table S1**. Selected bond lengths [Å] in ethers **3**, **4** and **5** determined by XRD data.

| **Bond lengths** | | 3A | 3B | **4** | 5A | 5B |
|---|---|---|---|---|---|---|
| 8a | 4a | 1.397(2) | 1.397(2) | 1.399(2) | 1.396(2) | 1.395(2) |
| 4a | 5 | 1.404(2) | 1.406(2) | 1.406(2) | 1.408(2) | 1.407(2) |
| 5 | 6 | 1.390(3) | 1.391(3) | 1.393(2) | 1.395(2) | 1.395(2) |
| 6 | 7 | 1.401(2) | 1.401(2) | 1.397(2) | 1.404(2) | 1.404(2) |
| 7 | 8 | 1.399(2) | 1.400(2) | 1.401(2) | 1.402(2) | 1.405(2) |
| 8 | 8a | 1.404(3) | 1.403(3) | 1.402(2) | 1.397(2) | 1.397(2) |
| 8 | 8b | 1.508(2) | 1.510(3) | 1.508(2) | 1.514(2) | 1.511(2) |
| 5 | 5a | 1.508(2) | 1.508(2) | 1.508(2) | 1.510(2) | 1.510(2) |
| 7 | 7a | 1.507(3) | 1.508(3) | 1.508(2) | 1.508(2) | 1.506(2) |
| O2 | 6 | 1.403(2) | 1.405(2) | 1.399(2) | 1.390(1) | 1.391(1) |
| O1 | 2 | 1.451(3) | 1.453(3) | 1.453(2) | 1.450(2) | 1.453(2) |
| O1 | 8a | 1.380(2) | 1.379(2) | 1.383(2) | 1.381(1) | 1.382(1) |
| O2 | 2b | 1.517(2) | 1.521(4) | 1.523(2) | 1.521(2) | 1.525(2) |
| O2 | 2a | 1.525(4) | 1.522(2) | 1.519(2) | 1.517(2) | 1.517(2) |
| O2 | C1'/Si | 1.427(7) | 1.418(3) | 1.426(2) | 1.666(1) | 1.667(1) |

**Table S2.** Selected bond angles [°] in ethers **3**, **4** and **5** determined by XRD data.

| Bond angle | | | **3**A | **3**B | **4** | **5**A | **5**B |
|---|---|---|---|---|---|---|---|
| 4a | 5 | 6 | 118.5(2) | 118.9(2) | 118.8(1) | 119.1(1) | 119.2(1) |
| 5 | 6 | 7 | 122.5(2) | 122.3(2) | 122.4(1) | 121.6(1) | 121.8(1) |
| 6 | 7 | 8 | 118.9(2) | 118.8(2) | 118.9(1) | 118.9(1) | 118.7(1) |
| 7 | 8 | 8a | 118.6(2) | 119.0(2) | 118.9(1) | 119.2(1) | 119.2(1) |
| 8 | 8a | 4a | 122.0(2) | 121.8(2) | 121.9(1) | 121.8(1) | 121.9(1) |
| 8a | 4a | 5 | 119.0(2) | 119.1(2) | 119.0(1) | 119.0(1) | 118.9(1) |
| 6 | 5 | 5a | 121.2(2) | 121.4(2) | 120.9(1) | 120.9(1) | 120.9(1) |
| 6 | 7 | 7a | 120.3(2) | 120.7(2) | 120.8(1) | 120.8(1) | 120.9(1) |
| 7 | 8 | 8b | 121.5(2) | 121.5(2) | 120.7(1) | 121.6(1) | 121.6(1) |
| 8 | 7 | 7a | 120.8(2) | 120.5(2) | 120.2(1) | 120.3(1) | 120.5(1) |
| 8b | 8 | 8a | 119.9(2) | 119.5(2) | 120.4(1) | 119.1(1) | 119.2(1) |
| O2 | 6 | 7 | 118.6(2) | 119.2(2) | 119.0(1) | 119.1(1) | 118.8(1) |
| O2 | 6 | 5 | 118.8(2) | 118.3(2) | 118.6(1) | 119.2(1) | 119.3(1) |

**Table S3.** Selected torsion angles [°] in ethers **3**, **4** and **5** determined by XRD data.

| Torsion angle | | | | **4** | **3**A | **3**B | **5**A | **5**B |
|---|---|---|---|---|---|---|---|---|
| 4a | 8a | O1 | 2 | −21.3 | 16.9 | 14.2 | 19.7 | −18.5 |
| 8b | 8 | 7 | 7a | 2.5 | 0.6 | −3.5 | 2.3 | −2.7 |
| 8 | 8a | 4a | 5 | −1.0 | 6.5 | 2.7 | −3.7 | 3.4 |
| 8a | 4a | 5 | 6 | 2.6 | −1.9 | −1.5 | −0.8 | 1.1 |
| 4a | 5 | 6 | 7 | −2.3 | −3.9 | −1.7 | 5.4 | −5.7 |
| 5 | 6 | 7 | 8 | 0.1 | 5.1 | 3.5 | −5.5 | 5.8 |
| 6 | 7 | 8 | 8a | 1.6 | −0.5 | −2.3 | 1.0 | −1.2 |
| 5 | 6 | 7 | 7a | 179.2 | −175.4 | 174.8 | 172.7 | −172.5 |
| 5a | 5 | 6 | 7 | 177.5 | 175.6 | 175.8 | −174.2 | 174.4 |
| 8b | 8 | 8a | 4a | 178.8 | 174.2 | 178.8 | −176 | 176.4 |
| 8a | 4a | 5 | 5a | −177.1 | 178.7 | −179 | 178.8 | −179 |
| O2 | 6 | 7 | 7a | −2.2 | 0.9 | −0.4 | −3.5 | 3.4 |
| O2 | 6 | 5 | 5a | −1.1 | −0.7 | 1.3 | 2.0 | −1.5 |

**Table S4**. The bonds length comparison in ethers **4**, **3**A-B and **5**A-B and differences in bond lengthens between **4** and ethers **3** and **5** with 3σ values).

| **Bond** | | **4** | **3A** | **3B** | **5A** | **5B** | Differences in bond length | | | | | | | |
|---|---|---|---|---|---|---|---|---|---|---|---|---|---|---|
| | | Length | | | | | **4 - 3A** | **(3σ)** | **4 - 3B** | **(3σ)** | **4 - 5A** | (3σ) | **4 - 5B** | (3σ) |
| C8a | C4a | 1.399(2) | 1.397(2) | 1.397(2) | 1.396(2) | 1.395(2) | 0.002 | 0.008 | 0.002 | 0.008 | 0.003 | 0.008 | 0.004 | 0.008 |
| C4a | C5 | 1.406(2) | 1.404(2) | 1.406(2) | 1.408(2) | 1.407(2) | 0.002 | 0.008 | 0 | 0.008 | −0.002 | 0.008 | −0.001 | 0.008 |
| C5 | C6 | 1.393(2) | 1.390(3) | 1.391(3) | 1.395(2) | 1.395(2) | 0.003 | 0.011 | 0.002 | 0.011 | −0.002 | 0.008 | −0.002 | 0.008 |
| **C6** | **C7** | **1.397(2)** | **1.401(2)** | **1.401(2)** | **1.404(2)** | **1.404(2)** | **−0.004** | **0.008** | **−0.004** | **0.008** | **−0.007** | **0.008** | **−0.007** | **0.008** |
| C7 | C8 | 1.401(2) | 1.399(2) | 1.400(2) | 1.402(2) | 1.405(2) | 0.002 | 0.008 | 0.001 | 0.008 | −0.001 | 0.008 | −0.004 | 0.008 |
| C8 | C8a | 1.402(2) | 1.404(3) | 1.403(3) | 1.397(2) | 1.397(2) | −0.002 | 0.011 | −0.001 | 0.011 | 0.005 | 0.008 | 0.005 | 0.008 |
| C8 | C8b | 1.508(2) | 1.508(2) | 1.510(3) | 1.514(2) | 1.511(2) | 0 | 0.008 | −0.002 | 0.011 | −0.006 | 0.008 | −0.003 | 0.008 |
| C5 | C5a | 1.508(2) | 1.508(2) | 1.508(2) | 1.510(2) | 1.510(2) | 0 | 0.008 | 0 | 0.008 | −0.002 | 0.008 | −0.002 | 0.008 |
| C7 | C7a | 1.508(2) | 1.507(3) | 1.508(3) | 1.508(2) | 1.506(2) | 0.001 | 0.011 | 0 | 0.011 | 0 | 0.008 | 0.002 | 0.008 |
| **C6** | **O2** | **1.399(2)** | **1.403(2)** | **1.405(2)** | **1.390(1)** | **1.391(1)** | −0.004 | 0.008 | −0.006 | 0.008 | **0.009** | **0.007** | **0.008** | **0.007** |
| O1 | C2 | 1.453(2) | 1.451(3) | 1.453(3) | 1.450(2) | 1.453(2) | 0.002 | 0.011 | 0 | 0.011 | 0.003 | 0.008 | 0 | 0.008 |
| O1 | C8a | 1.383(2) | 1.380(2) | 1.379(2) | 1.381(1) | 1.382(1) | 0.003 | 0.008 | 0.004 | 0.008 | 0.002 | 0.007 | 0.001 | 0.007 |
| C2 | C2b | 1.523(2) | 1.525(4) | 1.521(4) | 1.521(2) | 1.525(2) | −0.002 | 0.022 | 0.002 | 0.013 | 0.002 | 0.008 | −0.002 | 0.008 |
| C2 | C2a | 1.519(2) | 1.517(2) | 1.522(2) | 1.517(2) | 1.517(2) | 0.002 | 0.008 | −0.003 | 0.008 | 0.002 | 0.008 | 0.002 | 0.008 |

**Table S5**. The bonds length comparison between 4-methoxy−2,3,5,6-tetramethylphenol (4MTP, CCDC MOPHLA) and ethers **4**, **3**A-B and **5**A-B.

| **bond** | | **4PMT** | **4** | **3A** | **3B** | **5A** | **5B** | **4−4PMT** | **3A−4PMT** | **3B−4PMT** | **5A−4PMT** | **5B−4PMT** |
|---|---|---|---|---|---|---|---|---|---|---|---|---|
| **C8a** | **C4a** | **1.382** | **1.399** | **1.397** | **1.397** | **1.396** | **1.395** | **0.017** | **0.015** | **0.015** | **0.014** | **0.013** |
| **C4A** | **C5** | **1.394** | **1.406** | **1.404** | **1.406** | **1.408** | **1.407** | **0.012** | **0.010** | **0.012** | **0.014** | **0.013** |
| C5 | C6 | 1.390 | 1.393 | 1.39 | 1.391 | 1.395 | 1.395 | 0.003 | 0.000 | 0.001 | 0.005 | 0.005 |
| **C6** | **C7** | **1.387** | **1.397** | **1.401** | **1.401** | **1.404** | **1.404** | **0.010** | **0.014** | **0.014** | **0.017** | **0.017** |
| C7 | C8 | 1.398 | 1.401 | 1.399 | 1.4 | 1.402 | 1.405 | 0.003 | 0.001 | 0.002 | 0.004 | 0.007 |
| **C8** | **C8a** | **1.387** | **1.402** | **1.404** | **1.403** | **1.397** | **1.397** | **0.015** | **0.017** | **0.016** | **0.010** | **0.010** |
| C8 | C8a | 1.509 | 1.508 | 1.508 | 1.51 | 1.514 | 1.511 | −0.001 | −0.001 | 0.001 | 0.005 | 0.002 |
| C5 | C5a | 1.504 | 1.508 | 1.508 | 1.508 | 1.51 | 1.51 | 0.004 | 0.004 | 0.004 | 0.006 | 0.006 |
| C7 | C7a | 1.508 | 1.508 | 1.507 | 1.508 | 1.508 | 1.506 | 0.000 | −0.001 | 0.000 | 0.000 | −0.002 |
| C6 | O2 | 1.409 | 1.399 | 1.403 | 1.405 | 1.39 | 1.391 | −0.010 | −0.006 | −0.004 | −0.019 | −0.018 |
| O1 | C2 | 1.443 | 1.453 | 1.451 | 1.453 | 1.45 | 1.453 | 0.010 | 0.008 | 0.010 | 0.007 | 0.010 |
| **O1** | **C8a** | **1.409** | **1.383** | **1.38** | **1.379** | **1.381** | **1.382** | **−0.026** | **−0.029** | **−0.030** | **−0.028** | **−0.027** |

**Tabela S6**. Data for ether **3** from WinNMR.

| Temp. [K] | **165.0** | **170.5** | **172.5** | **174.0** | **176.0** | **177.6** | **179.2** | **182.0** | **184.5** | **193.0** | **219.7** |
|---|---|---|---|---|---|---|---|---|---|---|---|
| $\Delta G^{\#}_{(ab)}$ [kcal/mol] | 8.56 | 8.56 | 8.54 | 8.53 | 8.46 | 8.46 | 8.43 | 8.39 | 8.39 | 8.36 | 8.14 |
| 1/T [x10$^{-2}$] | 0.606 | 0.586 | 0.579 | 0.574 | 0.568 | 0.563 | 0.557 | 0.549 | 0.542 | 0.518 | 0.455 |
| k*ab* | 15.68 | 37.75 | 55.189 | 70.451 | 116.147 | 144.011 | 195.149 | 315. | 437.950 | 1360. | 36800 |
| ln*k* | 2.752 | 3.631 | 4.011 | 4.255 | 4.755 | 4.97 | 5.274 | 5.753 | 6.082 | 7.215 | 10.513 |

Average $\Delta G^{\#}_{(ab)}$ = 8.44 kcal·mol$^{-1}$; std. dev. = 0.42 kcal·mol$^{-1}$

**Table S7.** Energies [kJ/mol], relative energies [kJ/mol] and calculated NMR isotropic chemical shift values [ppm] of the studied conformations of 3.

| Conformation symbol | | a | b | c | d | e | f | g | h | i | j |
|---|---|---|---|---|---|---|---|---|---|---|---|
| Relative energy [kJ/mol] | | 0.00 | 0.00 | 0.00 | 0.00 | 0.00 | 19.86 | 20.09 | 26.25 | 30.74 | 30.74 |
| Energy [kJ/mol] | | −2513941.28 | −2513941.28 | −2513941.28 | −2513941.28 | −2513941.28 | −2513921.41 | −2513921.19 | −2513915.03 | 2513910.54 | 2513910.54 |
| Atom number | Exp. | | | | | GIAO | | | | | |
| 2 | 72.6 | 73.65 | 73.64 | 73.65 | 73.65 | 73.65 | 73.53 | 73.53 | 73.95 | 73.49 | 73.49 |
| 2a | 26.9 | 25.85 | 25.85 | 25.85 | 25.85 | 25.85 | 25.78 | 25.8 | 25.83 | 25.72 | 25.72 |
| 2b | 26.8 | 27.43 | 27.48 | 27.43 | 27.43 | 27.43 | 27.38 | 27.48 | 27.47 | 27.53 | 27.52 |
| 3 | 32.9 | 30.8 | 30.8 | 30.8 | 30.8 | 30.8 | 30.85 | 30.9 | 30.8 | 30.82 | 30.82 |
| 4 | 21.1 | 19.66 | 19.66 | 19.66 | 19.67 | 19.67 | 19.7 | 19.67 | 19.83 | 19.62 | 19.62 |
| 4a | 117 | 118.48 | 118.48 | 118.48 | 118.48 | 118.48 | 118.19 | 119.15 | 117.25 | 118.86 | 118.86 |
| 5 | 126.4 | 127.05 | 126.04 | 126.05 | 126.04 | 126.05 | 132.79 | 130.97 | 124.82 | 124.09 | 124.1 |
| 5a | 11.9 | 9.52 | 9.52 | 9.52 | 9.52 | 9.57 | 9.75 | 8.51 | 9.15 | 7.74 | 7.74 |
| 6 | 147.3 | 148.38 | 148.38 | 148.38 | 148.39 | 148.38 | 151.03 | 150.68 | 149.06 | 148.38 | 148.38 |
| 7 | 128.2 | 129.42 | 129.42 | 129.42 | 129.43 | 129.42 | 129.45 | 131.86 | 130.89 | 133.16 | 133.16 |
| 7a | 13.8 | 13.35 | 13.35 | 13.35 | 13.35 | 13.45 | 14.05 | 15.5 | 12.87 | 14.56 | 14.56 |
| 8 | 122.6 | 124.46 | 124.46 | 124.46 | 124.45 | 124.46 | 124.96 | 123.92 | 125.5 | 124.01 | 124.01 |
| 8b | 12.9 | 12.45 | 12.33 | 12.34 | 12.34 | 12.44 | 12.38 | 12.32 | 12.1 | 12.19 | 12.19 |
| 8a | 148 | 149.75 | 149.74 | 149.75 | 149.74 | 149.75 | 149.59 | 149.56 | 149.47 | 149.52 | 149.52 |
| 1' | 103.7 | 104.95 | 104.94 | 104.95 | 104.95 | 104.95 | 102.11 | 102.49 | 103.53 | 103.76 | 103.75 |
| 2' | 31.3 | 30.67 | 30.67 | 30.67 | 30.67 | 30.67 | 29.09 | 29.17 | 31.06 | 24.22 | 24.22 |
| 3' | 21.2 | 21.5 | 21.5 | 21.5 | 21.5 | 21.5 | 16.49 | 16.55 | 21.3 | 13.84 | 13.85 |
| 4' | 25.2 | 24.11 | 24.2 | 24.1 | 24.11 | 24.2 | 24.52 | 24.56 | 23.88 | 20.24 | 20.24 |

| | | | | | | | | | | | |
|---|---|---|---|---|---|---|---|---|---|---|---|
| 5' | 65.1 | 65.45 | 65.45 | 65.44 | 65.45 | 65.45 | 60.93 | 61 | 65.87 | 56.2 | 56.2 |
| | | | | | | Exp. - GIAO | | | | | |
| 2 | 72.6 | −1.05 | −1.04 | −1.05 | −1.05 | −1.05 | −0.93 | −0.93 | −1.35 | −0.89 | −0.89 |
| 2a | 26.9 | 1.05 | 1.05 | 1.05 | 1.05 | 1.05 | 1.12 | 1.1 | 1.07 | 1.18 | 1.18 |
| 2b | 26.8 | −0.63 | −0.68 | −0.63 | −0.63 | −0.63 | −0.58 | −0.68 | −0.67 | −0.73 | −0.72 |
| 3 | 32.9 | 2.1 | 2.1 | 2.1 | 2.1 | 2.1 | 2.05 | 2 | 2.1 | 2.08 | 2.08 |
| 4 | 21.1 | 1.44 | 1.44 | 1.44 | 1.43 | 1.43 | 1.4 | 1.43 | 1.27 | 1.48 | 1.48 |
| 4a | 117 | −1.48 | −1.48 | −1.48 | −1.48 | −1.48 | −1.19 | −2.15 | −0.25 | −1.86 | −1.86 |
| 5 | 126.4 | −0.65 | 0.36 | 0.35 | 0.36 | 0.35 | −6.39 | −4.57 | 1.58 | 2.31 | 2.3 |
| 5a | 11.9 | 2.38 | 2.38 | 2.38 | 2.38 | 2.33 | 2.15 | 3.39 | 2.75 | 4.16 | 4.16 |
| 6 | 147.3 | −1.08 | −1.08 | −1.08 | −1.09 | −1.08 | −3.73 | −3.38 | −1.76 | −1.08 | −1.08 |
| 7 | 128.2 | −1.22 | −1.22 | −1.22 | −1.23 | −1.22 | −1.25 | −3.66 | −2.69 | −4.96 | −4.96 |
| 7a | 13.8 | 0.45 | 0.45 | 0.45 | 0.45 | 0.35 | −0.25 | −1.7 | 0.93 | −0.76 | −0.76 |
| 8 | 122.6 | −1.86 | −1.86 | −1.86 | −1.85 | −1.86 | −2.36 | −1.32 | −2.9 | −1.41 | −1.41 |
| 8b | 12.9 | 0.45 | 0.57 | 0.56 | 0.56 | 0.46 | 0.52 | 0.58 | 0.8 | 0.71 | 0.71 |
| 8a | 148 | −1.75 | −1.74 | −1.75 | −1.74 | −1.75 | −1.59 | −1.56 | −1.47 | −1.52 | −1.52 |
| 1' | 103.7 | −1.25 | −1.24 | −1.25 | −1.25 | −1.25 | 1.59 | 1.21 | 0.17 | −0.06 | −0.05 |
| 2' | 31.3 | 0.63 | 0.63 | 0.63 | 0.63 | 0.63 | 2.21 | 2.13 | 0.24 | 7.08 | 7.08 |
| 3' | 21.2 | −0.3 | −0.3 | −0.3 | −0.3 | −0.3 | 4.71 | 4.65 | −0.1 | 7.36 | 7.35 |
| 4' | 25.2 | 1.09 | 1 | 1.1 | 1.09 | 1 | 0.68 | 0.64 | 1.32 | 4.96 | 4.96 |
| 5' | 65.1 | −0.35 | −0.35 | −0.34 | −0.35 | −0.35 | 4.17 | 4.1 | −0.77 | 8.9 | 8.9 |

| | | | | | | | | | | |
|---|---|---|---|---|---|---|---|---|---|---|
| Conformation symbol | k | l | m | n | o | p | q | r | s | t |
| Relative energy [kJ/mol] | 41.41 | 41.41 | 41.41 | 41.41 | 41.41 | 41.41 | 41.41 | 41.41 | 41.41 | 41.41 |
| Energy | −2513900 | −2513900 | −2513900 | −2513900 | −2513900 | −2513900 | −2513900 | −2513900 | −2513900 | −2513900 |

[kJ/mol]

| Atom number | Exp. | GIAO | | | | | | | | | |
|---|---|---|---|---|---|---|---|---|---|---|---|
| 2 | 72.6 | 73.66 | 73.66 | 73.66 | 73.66 | 73.66 | 73.66 | 73.66 | 73.66 | 73.77 | 73.66 |
| 2a | 26.9 | 25.86 | 25.86 | 25.86 | 25.85 | 25.86 | 25.86 | 25.86 | 25.86 | 25.86 | 25.86 |
| 2b | 26.8 | 27.45 | 27.44 | 27.44 | 27.44 | 27.44 | 27.45 | 27.44 | 27.44 | 27.44 | 27.44 |
| 3 | 32.9 | 30.8 | 30.8 | 30.8 | 30.8 | 30.8 | 30.8 | 30.8 | 30.8 | 30.8 | 30.8 |
| 4 | 21.1 | 19.67 | 19.67 | 19.7 | 19.67 | 19.67 | 19.67 | 19.67 | 19.67 | 19.67 | 19.67 |
| 4a | 117 | 118.52 | 118.6 | 118.52 | 118.52 | 118.52 | 118.52 | 118.52 | 118.51 | 118.52 | 118.52 |
| 5 | 126.4 | 127.47 | 127.47 | 127.47 | 127.47 | 127.47 | 127.47 | 127.46 | 127.47 | 127.47 | 127.46 |
| 5a | 11.9 | 9.57 | 9.57 | 9.57 | 9.57 | 9.57 | 9.56 | 9.57 | 9.57 | 9.57 | 9.57 |
| 6 | 147.3 | 148.37 | 148.37 | 148.37 | 148.37 | 148.37 | 148.37 | 148.37 | 148.47 | 148.37 | 148.37 |
| 7 | 128.2 | 129.4 | 129.4 | 129.4 | 129.4 | 129.4 | 129.4 | 129.4 | 129.4 | 129.4 | 129.4 |
| 7a | 13.8 | 13.01 | 13 | 13 | 13.01 | 13.01 | 13 | 13 | 13.01 | 13 | 13 |
| 8 | 122.6 | 124.43 | 124.43 | 124.43 | 124.44 | 124.43 | 124.43 | 124.43 | 124.43 | 124.43 | 124.43 |
| 8b | 12.9 | 12.3 | 12.3 | 12.29 | 12.29 | 12.3 | 12.29 | 12.3 | 12.29 | 12.29 | 12.3 |
| 8a | 148 | 149.76 | 149.76 | 149.76 | 149.76 | 149.76 | 149.76 | 149.76 | 149.75 | 149.76 | 149.76 |
| 1' | 103.7 | 101.42 | 101.42 | 101.42 | 101.42 | 101.42 | 101.43 | 101.42 | 101.42 | 101.42 | 101.41 |
| 2' | 31.3 | 28.34 | 28.43 | 28.34 | 28.34 | 28.34 | 28.33 | 28.34 | 28.34 | 28.34 | 28.34 |
| 3' | 21.2 | 16.35 | 16.35 | 16.35 | 16.45 | 16.35 | 16.35 | 16.35 | 16.35 | 16.35 | 16.35 |
| 4' | 25.2 | 19.53 | 19.53 | 19.53 | 19.53 | 19.53 | 19.52 | 19.53 | 19.53 | 19.53 | 19.53 |
| 5' | 65.1 | 62.63 | 62.63 | 62.63 | 62.63 | 62.63 | 62.63 | 62.63 | 62.63 | 62.63 | 62.63 |
| | | Exp. - GIAO | | | | | | | | | |
| 2 | 72.6 | −1.06 | −1.06 | −1.06 | −1.06 | −1.06 | −1.06 | −1.06 | −1.06 | −1.17 | −1.06 |
| 2a | 26.9 | 1.04 | 1.04 | 1.04 | 1.05 | 1.04 | 1.04 | 1.04 | 1.04 | 1.04 | 1.04 |
| 2b | 26.8 | −0.65 | −0.64 | −0.64 | −0.64 | −0.64 | −0.65 | −0.64 | −0.64 | −0.64 | −0.64 |
| 3 | 32.9 | 2.1 | 2.1 | 2.1 | 2.1 | 2.1 | 2.1 | 2.1 | 2.1 | 2.1 | 2.1 |
| 4 | 21.1 | 1.43 | 1.43 | 1.4 | 1.43 | 1.43 | 1.43 | 1.43 | 1.43 | 1.43 | 1.43 |

| | | | | | | | | | | | |
|---|---|---|---|---|---|---|---|---|---|---|---|
| 4a | 117 | −1.52 | −1.6 | −1.52 | −1.52 | −1.52 | −1.52 | −1.52 | −1.51 | −1.52 | −1.52 |
| 5 | 126.4 | −1.07 | −1.07 | −1.07 | −1.07 | −1.07 | −1.07 | −1.06 | −1.07 | −1.07 | −1.06 |
| 5a | 11.9 | 2.33 | 2.33 | 2.33 | 2.33 | 2.33 | 2.34 | 2.33 | 2.33 | 2.33 | 2.33 |
| 6 | 147.3 | −1.07 | −1.07 | −1.07 | −1.07 | −1.07 | −1.07 | −1.07 | −1.17 | −1.07 | −1.07 |
| 7 | 128.2 | −1.2 | −1.2 | −1.2 | −1.2 | −1.2 | −1.2 | −1.2 | −1.2 | −1.2 | −1.2 |
| 7a | 13.8 | 0.79 | 0.8 | 0.8 | 0.79 | 0.79 | 0.8 | 0.8 | 0.79 | 0.8 | 0.8 |
| 8 | 122.6 | −1.83 | −1.83 | −1.83 | −1.84 | −1.83 | −1.83 | −1.83 | −1.83 | −1.83 | −1.83 |
| 8b | 12.9 | 0.6 | 0.6 | 0.61 | 0.61 | 0.6 | 0.61 | 0.6 | 0.61 | 0.61 | 0.6 |
| 8a | 148 | −1.76 | −1.76 | −1.76 | −1.76 | −1.76 | −1.76 | −1.76 | −1.75 | −1.76 | −1.76 |
| 1' | 103.7 | 2.28 | 2.28 | 2.28 | 2.28 | 2.28 | 2.27 | 2.28 | 2.28 | 2.28 | 2.29 |
| 2' | 31.3 | 2.96 | 2.87 | 2.96 | 2.96 | 2.96 | 2.97 | 2.96 | 2.96 | 2.96 | 2.96 |
| 3' | 21.2 | 4.85 | 4.85 | 4.85 | 4.75 | 4.85 | 4.85 | 4.85 | 4.85 | 4.85 | 4.85 |
| 4' | 25.2 | 5.67 | 5.67 | 5.67 | 5.67 | 5.67 | 5.68 | 5.67 | 5.67 | 5.67 | 5.67 |
| 5' | 65.1 | 2.47 | 2.47 | 2.47 | 2.47 | 2.47 | 2.47 | 2.47 | 2.47 | 2.47 | 2.47 |

| Conformation symbol | | u | v | w | x | y | z |
|---|---|---|---|---|---|---|---|
| Relative energy [kJ/mol] | | 42.78 | 42.78 | 42.78 | 42.78 | 42.78 | 42.78 |
| Energy [kJ/mol] | | −2513899 | −2513899 | −2513899 | −2513899 | −2513899 | −2513899 |
| Atom number | Exp. | | | GIAO | | | |
| 2 | 72.6 | 73.52 | 73.52 | 73.52 | 73.52 | 73.52 | 73.52 |
| 2a | 26.9 | 25.75 | 25.75 | 25.75 | 25.75 | 25.75 | 25.75 |
| 2b | 26.8 | 27.49 | 27.48 | 27.48 | 27.48 | 27.48 | 27.48 |
| 3 | 32.9 | 30.78 | 30.77 | 30.77 | 30.77 | 30.77 | 30.77 |
| 4 | 21.1 | 19.47 | 19.47 | 19.47 | 19.37 | 19.47 | 19.47 |
| 4a | 117 | 118.63 | 118.63 | 118.63 | 118.63 | 118.63 | 118.63 |
| 5 | 126.4 | 123.69 | 123.69 | 123.69 | 123.69 | 123.69 | 123.69 |
| 5a | 11.9 | 7.79 | 7.79 | 7.8 | 7.79 | 7.79 | 7.79 |
| 6 | 147.3 | 148.26 | 148.25 | 148.25 | 148.25 | 148.26 | 148.25 |

| | | | | | | | |
|---|---|---|---|---|---|---|---|
| 7 | 128.2 | 133.78 | 133.78 | 133.79 | 133.79 | 133.78 | 133.79 |
| 7a | 13.8 | 15.1 | 15.11 | 15.09 | 15.1 | 15.11 | 15.11 |
| 8 | 122.6 | 124.15 | 124.15 | 124.14 | 124.15 | 124.15 | 124.15 |
| 8b | 12.9 | 11.92 | 11.92 | 11.91 | 11.92 | 11.92 | 11.92 |
| 8a | 148 | 149.39 | 149.39 | 149.39 | 149.39 | 149.39 | 149.39 |
| 1' | 103.7 | 102.14 | 102.15 | 102.15 | 102.13 | 102.14 | 102.15 |
| 2' | 31.3 | 28.38 | 28.37 | 28.38 | 28.37 | 28.38 | 28.37 |
| 3' | 21.2 | 16.33 | 16.33 | 16.43 | 16.33 | 16.35 | 16.33 |
| 4' | 25.2 | 19.46 | 19.46 | 19.46 | 19.46 | 19.46 | 19.46 |
| 5' | 65.1 | 62.48 | 62.48 | 62.48 | 62.48 | 62.49 | 62.48 |
| | | | Exp.-GIAO | | | | |
| 2 | 72.6 | −0.92 | −0.92 | −0.92 | −0.92 | −0.92 | −0.92 |
| 2a | 26.9 | 1.15 | 1.15 | 1.15 | 1.15 | 1.15 | 1.15 |
| 2b | 26.8 | −0.69 | −0.68 | −0.68 | −0.68 | −0.68 | −0.68 |
| 3 | 32.9 | 2.12 | 2.13 | 2.13 | 2.13 | 2.13 | 2.13 |
| 4 | 21.1 | 1.63 | 1.63 | 1.63 | 1.73 | 1.63 | 1.63 |
| 4a | 117 | −1.63 | −1.63 | −1.63 | −1.63 | −1.63 | −1.63 |
| 5 | 126.4 | 2.71 | 2.71 | 2.71 | 2.71 | 2.71 | 2.71 |
| 5a | 11.9 | 4.11 | 4.11 | 4.1 | 4.11 | 4.11 | 4.11 |
| 6 | 147.3 | −0.96 | −0.95 | −0.95 | −0.95 | −0.96 | −0.95 |
| 7 | 128.2 | −5.58 | −5.58 | −5.59 | −5.59 | −5.58 | −5.59 |
| 7a | 13.8 | −1.3 | −1.31 | −1.29 | −1.3 | −1.31 | −1.31 |
| 8 | 122.6 | −1.55 | −1.55 | −1.54 | −1.55 | −1.55 | −1.55 |
| 8b | 12.9 | 0.98 | 0.98 | 0.99 | 0.98 | 0.98 | 0.98 |
| 8a | 148 | −1.39 | −1.39 | −1.39 | −1.39 | −1.39 | −1.39 |
| 1' | 103.7 | 1.56 | 1.55 | 1.55 | 1.57 | 1.56 | 1.55 |
| 2' | 31.3 | 2.92 | 2.93 | 2.92 | 2.93 | 2.92 | 2.93 |
| 3' | 21.2 | 4.87 | 4.87 | 4.77 | 4.87 | 4.85 | 4.87 |

| | | | | | | | |
|---|---|---|---|---|---|---|---|
| 4' | 25.2 | 5.74 | 5.74 | 5.74 | 5.74 | 5.74 | 5.74 |
| 5' | 65.1 | 2.62 | 2.62 | 2.62 | 2.62 | 2.61 | 2.62 |
